# Targetable Pathways for Alleviating Mitochondrial Dysfunction in Neurodegeneration of Metabolic and Non-Metabolic Diseases

**DOI:** 10.3390/ijms222111444

**Published:** 2021-10-23

**Authors:** Lauren Elizabeth Millichap, Elisabetta Damiani, Luca Tiano, Iain P. Hargreaves

**Affiliations:** 1 Department of Life and Environmental Sciences, Polytechnic University of Marche, I-60131 Ancona, Italy; l.millichap@pm.univpm.it (L.E.M.); e.damiani@univpm.it (E.D.); l.tiano@univpm.it (L.T.); 2 School of Pharmacy, Liverpool John Moores University, Liverpool L3 5UA, UK

**Keywords:** mitochondrial dysfunction, oxidative stress, neurodegeneration, Parkinson’s disease, methylmalonic acidaemia, lysosomal storage disorders, mitochondrial biogenesis, mitophagy, antioxidant defenses, therapeutics

## Abstract

Many neurodegenerative and inherited metabolic diseases frequently compromise nervous system function, and mitochondrial dysfunction and oxidative stress have been implicated as key events leading to neurodegeneration. Mitochondria are essential for neuronal function; however, these organelles are major sources of endogenous reactive oxygen species and are vulnerable targets for oxidative stress-induced damage. The brain is very susceptible to oxidative damage due to its high metabolic demand and low antioxidant defence systems, therefore minimal imbalances in the redox state can result in an oxidative environment that favours tissue damage and activates neuroinflammatory processes. Mitochondrial-associated molecular pathways are often compromised in the pathophysiology of neurodegeneration, including the parkin/PINK1, Nrf2, PGC1α, and PPARγ pathways. Impairments to these signalling pathways consequently effect the removal of dysfunctional mitochondria, which has been suggested as contributing to the development of neurodegeneration. Mitochondrial dysfunction prevention has become an attractive therapeutic target, and there are several molecular pathways that can be pharmacologically targeted to remove damaged mitochondria by inducing mitochondrial biogenesis or mitophagy, as well as increasing the antioxidant capacity of the brain, in order to alleviate mitochondrial dysfunction and prevent the development and progression of neurodegeneration in these disorders. Compounds such as natural polyphenolic compounds, bioactive quinones, and Nrf2 activators have been reported in the literature as novel therapeutic candidates capable of targeting defective mitochondrial pathways in order to improve mitochondrial function and reduce the severity of neurodegeneration in these disorders.

## 1. Introduction 

**Figure 1:**
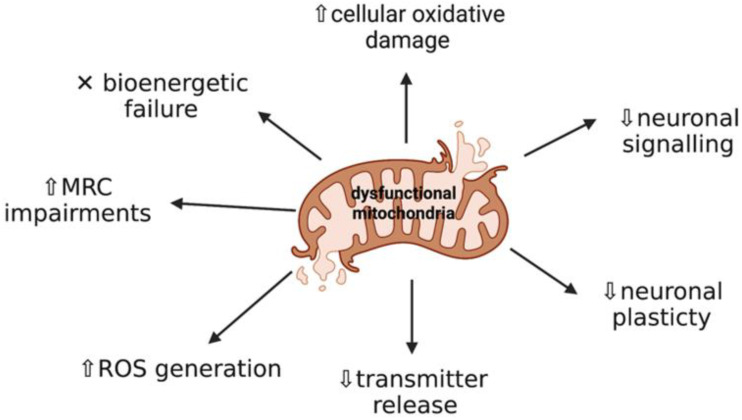
Mitochondrial dysfunction can lead to several deleterious consequences, contributing to the development and progression of neurodegeneration in a range of diseases. Failure to remove dysfunctional mitochondria can have profound effects on the central nervous system, leading to cellular distress and damage, ultimately leading to neuronal death. Up arrows represent increases and down arrows represent reductions (created using biorender.com, accessed on 17 June 2021). MRC—mitochondrial respiratory chain; ROS—reactive oxygen species.

Neurodegeneration is characterised by progressive deterioration of the neuronal structure and function, leading to cognitive impairments and dementia [1], and current evidence suggests that mitochondria have a fundamental role in the development of neurodegeneration [2]. Mitochondria are ubiquitous intracellular organelles that are the sites of aerobic metabolism, producing adenosine triphosphate (ATP), as well as being responsible for other processes including apoptosis regulation, cellular calcium homeostasis, and the generation of free radicals [3]. Mitochondria are sensitive to minor changes in the cellular redox status [4], therefore mitochondrial dysfunction can lead to several deleterious consequences such as oxidative stress (OS), secondary excitotoxicity, and activation of the mitochondrial permeability transition pore (mPTP). Progressive impairments to mitochondrial activity have been suggested to have a critical role in the pathogenesis of a range of diseases, including progressive neurodegenerative diseases, such as Parkinson’s disease, Alzheimer’s, disease and Amyotrophic Lateral Sclerosis, and metabolic diseases, including methylmalonic acidaemia, lysosomal storage disorders, and Friedreich’s ataxia [5] (Figure 1). 

Oxidative and nitrosative stress-related mitochondrial dysfunction has become a main focus of the pathogenic mechanisms that result in neuronal loss and neurodegeneration [6,7]. OS, defined by an imbalance between the excessive production of reactive oxygen species (ROS) and reactive nitrogen species (RNS) and a reduced cellular antioxidant capacity, can disrupt the normal function of cellular metabolic processes, leading to impairments in redox and energy production [8,9,10]. The cell contains a number of antioxidant defence mechanisms in order to neutralise ROS/RNS, including enzymatic antioxidants such as glutathione peroxidase (GPx), catalase (CAT), and superoxide dismutase (SOD), and non-enzymatic antioxidants such as glutathione (GSH), α-tocopherol (vitamin E), ascorbic acid (vitamin C), and natural flavonoids [11,12]. However, OS occurs if the generation of these highly reactive molecules overwhelms the cellular antioxidant capacity, therefore both enzymatic and non-enzymatic defences are no longer able to prevent ROS/RNS from causing oxidative damage to several cellular components, including lipids, protein, and DNA [13].

**Figure 2:**
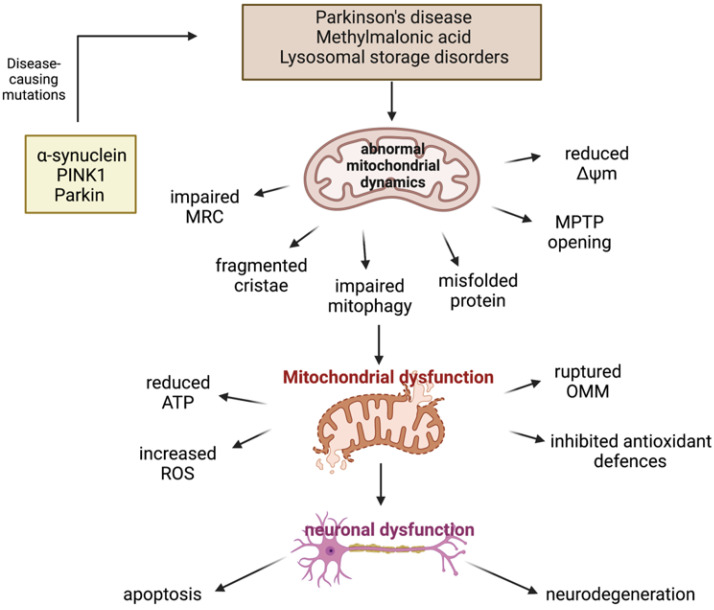
Putative common mechanisms leading to mitochondrial dysfunction and neurodegeneration in Parkinson’s disease, methylmalonic acidaemia, and lysosomal storage disorders. α-Synuclein, PINK1, and parkin are proteins frequently mutated in neurodegenerative disorders causing abnormal mitochondrial dynamics, including altered mitochondrial morphology, impaired MRC function and mitophagy, misfolded proteins, and reduced ΔΨm. These consequences result in mitochondrial dysfunction, triggering neuronal dysfunction that leads to neurodegeneration and neuronal death (created using biorender.com). PINK1—TEN-induced kinase 1; MRC—mitochondrial respiratory chain; MPTP—mitochondrial permeability transition pore; ΔΨm—mitochondrial membrane potential; ATP—adenosine triphosphate; ROS—reactive oxygen species; OMM—outer mitochondrial membrane.

The central nervous system (CNS) depends on efficient mitochondrial function [14] in order to maintain neuronal integrity and survival [15]. Mitochondria are the major source of endogenous ROS produced as a by-product from mitochondrial respiration, therefore the CNS is a very susceptible target for the accumulation and damage induced by ROS [16]. Additionally, mitochondrial-associated molecular pathways are often compromised [17] in the pathophysiology of neurodegeneration, including proteins frequently dysregulated in mitochondrial biogenesis [18] and mitophagy [19], consequently effecting the removal of dysfunctional mitochondria. Failure to remove defective mitochondria can cause cellular distress and damage [20], and has been suggested to be a cause of neuronal death leading to neurodegeneration (Figure 2) [17]. However, it is currently unknown whether oxidative damage and/or abnormal mitochondrial morphology occur early on in disease progression or whether they are caused by secondary manifestations of the disease pathophysiology [21].

New evidence has revealed that mitochondrial-targeted therapies represent important novel therapeutic candidates for restoration of the mitochondrial function by ameliorating OS and mitochondrial dysfunction in order to maintain neuronal cell integrity and function in disorders that involve neurodevelopment disruptions, including Parkinson’s disease, methylmalonic acidaemia, and lysosomal storage disorders [22]. These are highly prevalent disorders and mitochondrial dysfunction is a common theme among these three inheritable and non-communicable disorders [23]. Therefore, targeting dysfunctional mitochondria could be a potential therapeutic target in order to treat and/or prevent neurodegeneration in these disorders. Several compounds have been identified in the literature as being capable of targeting defective mitochondrial pathways in order to enhance mitochondrial function and reduce the consequences of OS, for example natural polyphenolic compounds, such as resveratrol and curcumin, capable of inducing mitochondrial biogenesis [24]; widely used FDA-approved drugs, such as dimethyl fumarate (DMF) [25], known to successfully target impaired mitophagy, and the Nrf2-antioxidant responsive element (Nrf2-ARE) pathway, an endogenous antioxidant defence system; and novel therapeutics, such as deubiquitinases (DUBs), modulating mitochondrial clearance via mitophagy [26]. 

The aim of this review will be to focus on common mechanisms leading to mitochondrial dysfunction and neurodegeneration in diseases representing non-metabolic neurodegenerative disorders, such as Parkinson’s disease, together with examples of rare metabolic diseases, such as methylmalonic acidaemia, together with more common metabolic diseases as exemplified by lysosomal storage disorders. This review will then discuss the various mitochondrial-associated molecular pathways that are frequently dysregulated in these disorders, including the parkin/PINK1, Nrf2, PGC1α, and PPARγ pathways, as well as outlining potential therapeutic strategies in order to alleviate the mitochondrial dysfunction and OS associated with neurodegeneration in these metabolic and non-metabolic disorders. 

## 2. Mitochondrial Dysfunction and Oxidative Stress: Key Events in Neurodegeneration 

Neurodegenerative and inherited metabolic diseases are a heterogenous group of disorders that can be inherited in an autosomal recessive pattern and be associated with neurological impairment, characterised by progressive loss of neurons in regions of the brain that compromise nervous system function [27,28]. The majority of disorders involving the CNS result in major neurodevelopment disruptions producing a wide variety of clinical manifestations, ranging from mild neurological signs, such as learning difficulties, to severe global encephalopathies [29]. Recent evidence has demonstrated that mitochondrial dysfunction and oxidative stress are important aetiological factors in the development and progression of neurodegeneration, resulting in alterations in the cellular redox state and injury to nervous system structural components, eventually leading to neuronal death. 

**Figure 3:**
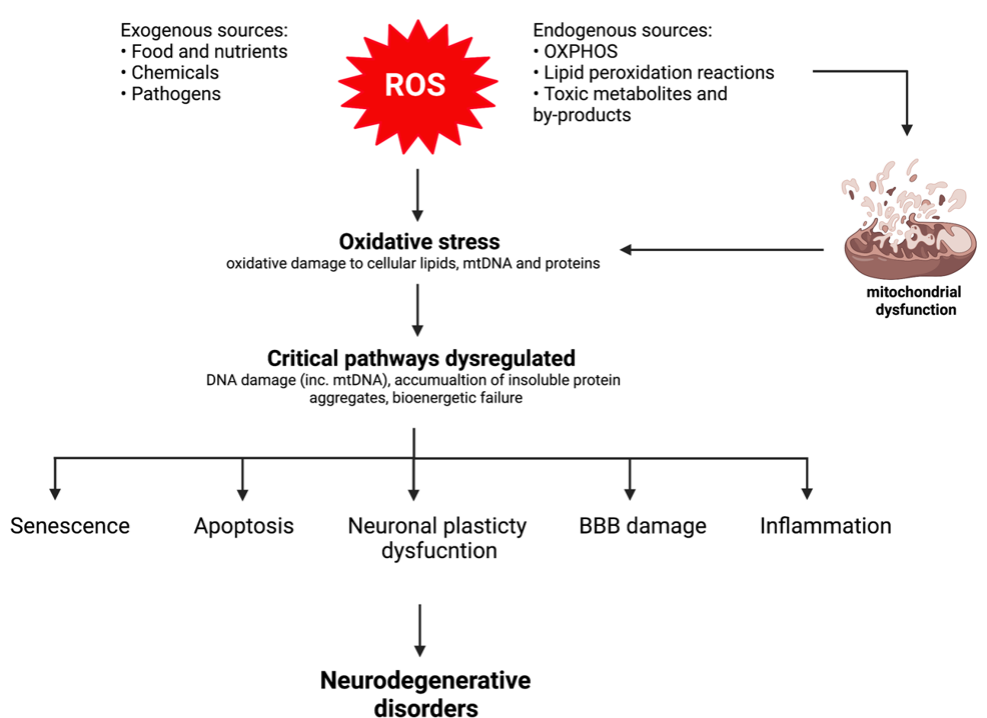
Mitochondrial dysfunction and oxidative stress are key events triggering neurodegeneration. Mitochondria are vulnerable targets for oxidative stress. ROS accumulation from mitochondria and endogenous and exogenous sources can cause oxidative damage to cellular components as they go unchallenged from diminished antioxidant defence systems. A chronic oxidation environment causes dysregulation of the critical pathways causing a lower gene expression of the antioxidant enzymes and transcription factors involved in mitochondria-dependent apoptosis. A combination of these factors results in neuronal apoptosis and senescence, triggering neurodegeneration (created using biorender.com). ROS—reactive oxygen species; OXPHOS—oxidative phosphorylation; mtDNA—mitochondrial DNA; BBB—blood−brain barrier.

Evidence of OS and secondary mitochondrial dysfunction have been hypothesised as key events triggering neurodegeneration (Figure 3) [30,31]. Firstly, OS-induced impairments to mitochondrial function contribute to cellular energy failure as a result of the overproduction of free radicals generated as part of the pathogenesis of disease [9]. ROS-induced mitochondrial impairments include mitochondrial DNA (mtDNA) damage leading to mutations in the mitochondrial genome, direct impairment to complexes in the mitochondrial respiratory chain (MRC), changes in mitochondrial membrane permeability and structure, and reduced efficiency of the mitochondrial defence systems [31]. Free radicals are produced under normal physiological conditions as the mitochondria contain numerous electron carriers producing ROS [6]; however, free radical-induced oxidative damage caused by the simultaneous accumulation of ROS/RNS and inefficient ROS protection exacerbates mitochondrial dysfunction. Moreover, other factors responsible for dysfunctional mitochondria include mitochondrial matrix enzymes, such as α-ketoglutarate dehydrogenase and pyruvate dehydrogenase, which are important ROS generators, and tricarboxylic acid (TCA) cycle enzymes, such as aconitase and α-ketoglutarate dehydrogenase, which are susceptible to ROS-mediated damage [32,33]. In addition, the accumulation of toxic molecules and by-products characteristic of certain neurodegenerative and metabolic diseases also contribute to mitochondrial dysfunction [30]. These changes are implicated in the pathogenesis of these disorders, and therefore facilitate neuronal dysfunction, triggering neurodegeneration [31].

The CNS depends on efficient mitochondria in order to function effectively, as the brain and neurons have high metabolic demands [6,14]. Under physiological conditions, ROS are essential for the development and function of neurons in small quantities, and the brain is able to effectively regulate oxygen consumption and redox capacity [34]. However, the brain has a high oxygen consumption rate; low antioxidant defence mechanisms [15], comprising of low levels of GSH and GPx and almost no CAT [6]; and a high concentration of polyunsaturated fatty acids, which are prone to oxidation [15], therefore meaning that the brain is very susceptible to oxidative injury. It is well-known that ROS accumulation increases the susceptibility of the cerebral tissue to damage, and free radicals are believed to trigger a cascade of molecular events resulting in altered brain morphology and increases in blood–brain barrier (BBB) permeability [34]. These alterations activate neuroinflammatory processes, favouring cellular injury and death. 

Furthermore, neurons are particularly more sensitive to the accumulation and damage induced by ROS, as neurons rely on mitochondria due to their inadequate glycolytic capacity, which means that they have a high rate of mitochondrial activity in order to fulfil their energetic requirements via oxidative phosphorylation (OXPHOS) [13,27]. This results in disruption of neuronal homeostasis, as mitochondria are the major source of endogenous ROS produced as a by-product of OXPHOS [16]. Therefore, OS-induced alterations in mitochondrial dynamics and reduced cellular energy status, as well as decreased neuronal plasticity, eventually contribute to the development of neurodegeneration [6].

## 3. Altered Signaling Pathways Leading to Mitochondrial Dysfunction in Neurological Disease 

### 3.1. Protein Function in Health

#### 3.1.1. Parkin and PINK1

Parkin and phosphatase and tensin homolog (PTEN)-induced kinase 1 (PINK1) are mitochondrial quality control regulators, stimulating the removal of damaged mitochondria [35]. Parkin is widely expressed in various tissues, including the brain, where this protein is equally expressed in several brain sub-regions and is most abundant in the substantia nigra [36]. Parkin functions as a cytosolic E3 ubiquitin ligase, ubiquitinating proteins targeted for signalling or proteasomal degradation [35]. Moreover, despite parkin being localised to the cytosol, it is an important protein for efficient mitochondrial function, as parkin recruitment from the cytosol to depolarised mitochondria is crucial for the selective autophagic removal (mitophagy) of damaged mitochondria, therefore maintaining a functional mitochondrial pool [35,37].

PINK1 is a mitochondrial serine/threonine kinase that is essential for the maintenance of mitochondrial homeostasis and has an important role within mitophagy. PINK1 exerts a neuroprotective effect and functions to protect dopaminergic neurons against OS-induced apoptosis, as it is capable of inhibiting ROS production by functioning as a mitochondrial serine/threonine (Ser/Thr) protein kinase [8,38,39]. The kinase activity of PINK1 has an essential role in the induction of mitophagy, as damaged mitochondria are removed following the activation of PINK1 and parkin [40]. Under healthy, physiological conditions, PINK1 accumulates at the outer mitochondrial membrane (OMM) of the damaged mitochondria in response to a reduction in mitochondrial membrane potential (ΔΨm) or misfolded protein accumulation, therefore initiating the translocation of parkin to the mitochondria. Once stabilised at the OMM, PINK1 phosphorylates ubiquitin at Ser65 in order to activate the ubiquitin ligase activity of parkin, and removes defective mitochondria via selective autophagy.

#### 3.1.2. Peroxisome Proliferator-Activated Receptor Gamma Coactivator 1-Alpha (PGC1α)

PGC1α, a transcriptional coactivator, is well-known as the master regulator of mitochondrial biogenesis and function, and is highly expressed in tissues with high energy demands and mitochondria-rich cells, such as neurons [24,41]. PGC1α regulates mitochondrial biogenesis by making mitochondria more bioenergetically competent, favouring enhanced mitochondrial biogenesis [42]. Mitochondrial mass is controlled via a dynamic equilibrium between degradation and biogenesis, and mitochondrial biogenesis is primarily regulated via the activation of PGC1α, leading to the activation of other transcription factors, including nuclear respiratory factors (NRF-1 and -2) and PPARs [24]. PGC1α interacts with NRF-1 and NRF-2 to stimulate their transcriptional activity via protein–protein interactions, resulting in an increased expression of mitochondrial transcription factor A (TFAM), subsequently stimulating mitochondrial biogenesis via the increased expression of intracellular ATP concentrations and OXPHOS [42]. Moreover, the expression of cellular antioxidant defences, including manganese superoxide dismutase (MnSOD/SOD2) and CAT, are upregulated via PGC1α in order to protect the cell from excessive mitochondrial ROS generation and dysfunction, and to prevent mitochondrial failure-associated cell death. PGC1α expression and activity are also activated via ROS/RNS. Furthermore, PGC1α modulates the key pathways required for neuronal function, and PGC1α activity in neurons may be involved in the regulation of neuronal mitochondrial density, as well as the cellular response to OS in order to reduce overall tissue damage [22,43]. Therefore, PGC1α may represent a key target for increasing healthy and functional mitochondrial pools [44].

#### 3.1.3. Peroxisome Proliferator-Activated Receptor Gamma (PPARγ)

PPARγ is a member of the peroxisome proliferator-activated receptors (PPARs), which are a nuclear receptor superfamily of ligand-inducible transcription factors compromising of three subtypes; PPARα, PPARγ, and PPARβ/δ [45]. The PPAR family has a major role in the regulation of energy homeostasis and metabolic function, and have distinctive tissue expression, however PPARγ is highly expressed in dopaminergic cells in the basal ganglia as well as in the midbrain [45,46]. The primary function of PPARγ is to regulate the expression of the gene networks involved in metabolism, metabolic homeostasis, and inflammation [47]. In addition, PPARγ activation is involved in cellular differentiation and proliferation, as well as having anti-apoptotic and anti-oxidant effects [46]. It is a key positive regulator of mitochondrial biogenesis and is able to restore the cellular redox environment via the activation of the PPARγ coactivator, PGC1α [44]. It has been demonstrated that mitochondrial biogenesis is mediated via PPARγ stimulation via the induction of PGC1α [48]. Studies have demonstrated that PPAR dysfunction is associated with CNS dysfunction and therefore demonstrates the key role that PPARγ has in maintaining nervous system integrity [46].

#### 3.1.4. Nuclear Factor Erythroid 2-Related Factor (Nrf2)

Nrf2, referred to as the master regulator of antioxidant detoxification and cell defence gene expression, is a transcription factor that plays a crucial role in protecting the cell against environmental stressors through a promoter sequence, known as the antioxidant-responsive element (ARE) [49]. The Nrf2-ARE pathway activates numerous cytoprotective genes including antioxidants and several transcription factors involved in mitochondrial biogenesis [50]. Mitochondrial biogenesis is thought to be induced via the translocation of Nrf2 to the nucleus where Nrf2 binds to the NRF1 promoter of ARE, activating TFAM, which directly drives mtDNA replication [51]. In addition, Nrf2 has important roles in cell survival, energy metabolism, and the anti-inflammatory response [49]. Under physiological conditions, Nrf2 is found in the cytosol where it is bound to Kelch-like ECH-associated protein 1 (Keap1), until it is activated via oxidants and electrophiles, resulting in the migration and accumulation of Nrf2 in the nucleus. Thus, increasing the expression of various antioxidants within the cell, including SOD1, GSH, and NADH quinone oxidoreductase (NQO1), therefore protecting the cell from OS events [38]. 

**Figure 4:**
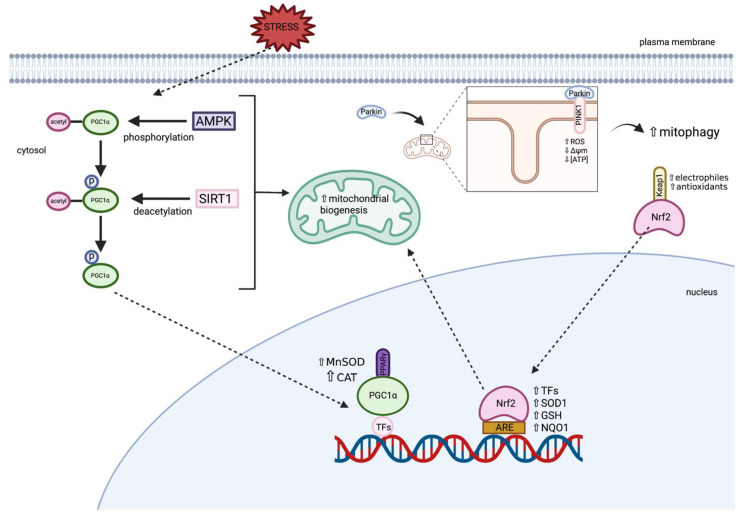
Signaling pathways involved in maintaining a healthy mitochondrial pool in health. PINK1 and parkin have protective roles in neurons and have essential roles in mitophagy. PINK1 accumulates at the outer mitochondrial membrane of damaged mitochondria, initiating the translocation of parkin from the cytosol to mitochondria, activating parkin and selecting damaged mitochondria for removal via mitophagy. The activation of PGC1α regulates mitochondrial biogenesis, leading to upregulation of several transcription factors and cellular antioxidants. PGC1α is directly regulated via AMPK phosphorylation and SIRT1-mediated deacetylation which induces mitochondrial biogenesis. PPARγ is a positive regulator of mitochondrial biogenesis and interaction between PPARγ and PGC1α allows PGC1α to interact with several transcription factors and restore the cellular redox environment by regulating the expression of cellular antioxidants, including MnSOD and CAT. Nrf2 is bound to Keap1 in the cytosol and migrates and accumulates in the nucleus upon activation by antioxidants and electrophiles. Nrf2 binds to the promoter sequence of the ARE where it upregulates the expression of several cellular antioxidants and transcription factors involved in mitochondrial biogenesis [Created using biorender.com]. PINK1—PTEN-induced kinase 1; ROS—reactive oxygen species; ΔΨm—mitochondrial membrane potential; ATP—adenosine triphosphate; Keap1—Kelch-like ECH-associated protein 1; Nrf2—nuclear factor erythroid 2-related factor; PGC1α—peroxisome proliferator-activated receptor gamma coactivator 1-alpha; AMPK—AMP-dependent kinase; SIRT1—silent mating type information regulation 2 homolog 1; ARE—antioxidant-responsive element; TF—transcription factor; SOD—superoxide dismutase; GSH—glutathione; NQO1—NADH quinone oxidoreductase; MnSOD—manganese superoxide dismutase; CAT—catalase; PPARγ—peroxisome proliferator-activated receptor gamma.

The signalling molecules involved in the maintenance of a healthy mitochondrial pool in health are outlined in Figure 4.

### 3.2. Non-Communicable Diseases

Non-communicable diseases (NCDs) are defined as chronic conditions that are non-infectious and non-transmissible, with the most common NCDs being related to the cardiovascular and nervous system [52]. Mitochondrial dysfunction, OS, and inflammation are common associations with NCDs and are suggestive of playing a critical role in disease pathogenesis of these disorders. Several lines of evidence have demonstrated that impaired mitochondrial function and dynamics are causative factors in several neurodegenerative diseases, and altered molecular pathways affecting mitochondrial function in these disorders are believed to be major risk factors in disease progression, as regulation of mitochondrial function is essential for neuronal signalling, plasticity, and transmitter release [50,53]. 

#### 3.2.1. Parkinson’s Disease

Parkinson’s disease (PD) is the second most prevalent neurodegenerative disorder after Alzheimer’s disease, and the most common movement disorder amongst the current population [54]. The clinical manifestations of the disease are characterised by clinical motor symptoms, including bradykinesia, resting tremor, rigidity, and postural instability, as well as non-motor symptoms, including cognitive deficits, depression, and autonomic and sensory dysfunction, eventually leading to near total immobility [54,55]. PD is characterised by two main pathological features, loss of dopaminergic neurons in the substantia nigra pars compacta (SNpc) of the midbrain and the development of intracytoplasmic inclusions, known as Lewy bodies, predominantly containing accumulated fibrillar α-synuclein in affected regions of the brain. These neuropathologies are thought to underlie the characteristic motor phenotype of PD patients, contributing to the development of neurodegeneration [53]. Although the aetiology of PD has not yet been fully elucidated, several human studies and animal models of PD have demonstrated that abnormal mitochondrial function and protein accumulation are key contributors in the pathogenesis of both sporadic and familial forms of PD, as well as OS, which contributes to the development of the two main pathogenic events [55]. Moreover, PD is frequently attributable to mutations in genes encoding for α-synuclein, parkin, and PINK1, and therefore can provide insights into the altered molecular pathways underlying neurodegeneration in PD. 

##### PINK1/Parkin Mutations and Mitochondrial Dysfunction

**Figure 5:**
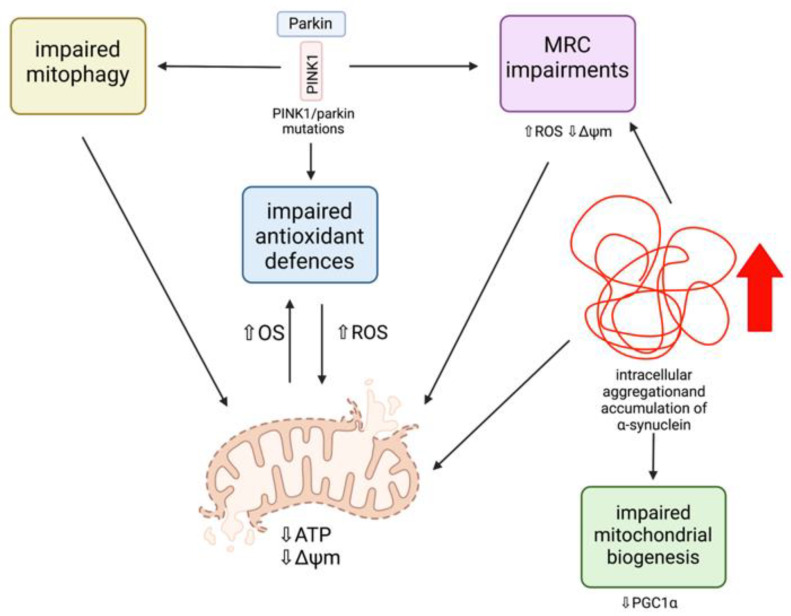
Mitochondrial dysfunction has a major role in the pathogenesis of Parkinson’s disease. Parkin/PINK1 mutations result in defective mitochondrial morphology, including MRC defects and impaired mitophagy. Parkin mutations can impair antioxidant defences, inducing oxidative stress and increasing ROS levels. α-Synuclein aggregation and accumulation (represented by up arrow) can increase oxidative stress resulting in increases in mitochondrial ROS production and mitochondrial fragmentation, altering the mitochondrial morphology. Intracellular α-synuclein aggregation impairs mitochondrial biogenesis via direct binding to the PGC1α promoter, downregulating the PGC1α expression. Both parkin/PINK1 mutations and α-synuclein accumulation cause reduced ΔΨm and ATP levels, leading to mitochondrial bioenergetic dysfunction and neurodegeneration (created using biorender.com). MRC—mitochondrial respiratory chain; ROS—reactive oxygen species; PGC1α—peroxisome proliferator-activated receptor gamma coactivator 1-alpha; ΔΨm—mitochondrial membrane potential; ATP—adenosine triphosphate; PINK1—PTEN-induced kinase 1; OS—oxidative stress.

Several lines of evidence have demonstrated that mitochondrial dysfunction has a primary role in the pathogenesis of PD, as many of the pathogenic mutations associated with PD are directly related to mitochondrial dysfunction (Figure 5) [22,35]. Parkin mutations are the most common cause of autosomal recessive PD, and are also the most sensitive to oxidative damage, and PINK1 mutations are the second most frequent cause of autosomal recessive early-onset PD [56]. In order to ensure cell survival, healthy neurons remove dysfunctional mitochondria efficiently via mitophagy as a quality control mechanism [37]. However, mutations resulting in a loss of function of both parkin and PINK1 result in the death of many cell types, including dopaminergic neurons, whose dysfunction is mainly responsible for the classical motor deficits of PD [57]. This is because these neurons have a reduced ability to remove dysfunctional mitochondria as a consequence of mutations in parkin and PINK1, preventing the recruitment of parkin to the mitochondria and therefore interfering with mitophagy efficiency [37]. This results in an accumulation of damaged mitochondria, making these cells vulnerable targets for OS and death [58]. Additionally, mitochondrial ROS production is significantly increased as a result of dysfunctional PINK1 and parkin [59]. A reduced ability to cleave PINK1 between Ala-103 and Phe-104 in order to generate the 53 kDA PINK1 protein, augments mitochondrial and cytoplasmic ROS production, in addition to significantly reducing basal ΔΨm [60]. Moreover, parkin has been recognised to conjugate both phosphorylated and unphosphorylated ubiquitin to the substrate located on the depolarized mitochondria [60]. However, if this mechanism is dysfunctional, an accumulation of abnormal mitochondria and an overproduction of ROS can occur.

Studies in *Drosophila melanogaster* first identified the role of parkin and PINK1 in the maintenance of the mitochondria [37]. PINK1 knockout (KO) *Drosophila* were found to have defects in mitochondrial morphology, including fragmented mitochondrial cristae, hypersensitivity to OS, MRC defects, and degeneration of dopaminergic neurons, which are mechanisms that have been suggested as contributing early on in the pathogenesis of PD [56,61,62]. In parkin KO *Drosophila*, the same phenotype was observed as in the PINK1 KO *Drosophila* as a result of these proteins operating in the same genetic pathway in order to maintain functional mitochondria [63]. Moreover, altered mitochondrial morphology and muscle degeneration in PINK1 KO *Drosophila* were salvaged via parkin overexpression, but this was not observed with PINK1 overexpression in parkin KO *Drosophila*, indicating that PINK1 is an upstream regulator of parkin function [56]. Similar to *Drosophila* models, PINK1 KO mice exhibit MRC defects within the striatum. A study carried out by Gispert et al. (2009) generated PINK1 deficient mice and identified characteristics similar to PD development in humans, including dopaminergic synapse dysfunction and protuberant mitochondrial dysfunction [61]. The study observed that PINK1 KO mice had impaired mitochondrial electron transport chain (ETC) complex I + III + IV activity within the brain, as well as reductions in ΔΨm and ATP generation, resulting in mitochondrial bioenergetic dysfunction. A reduction in ΔΨm below a certain threshold is often caused by uncouplers or OS, and is required for the activation of mitophagy, favouring the accumulation of PINK1 at the OMM [64,65]. However, PINK1 processing is incomplete in damaged mitochondria as a consequence of reduced ΔΨm. Consequently, a deficiency in PINK1 becomes more apparent under conditions of cellular stress, which may explain why dopaminergic neurons in PINK1-associated PD have an increased susceptibility to death, as these neurons are particularly vulnerable to increased levels of OS as a result of dopamine metabolism, and PINK1 KO causes increased ROS generation [38,61]. Dopamine and its oxidation by-products may contribute to increased ROS generation and mitochondrial respiration inhibition in PD, specifically the inactivation of mitochondrial electron transport chain (ETC) complex I, as these metabolites have been found to interact with the OXPHOS system [66,67,68]. In isolated brain mitochondria from a rat model of PD, dopamine was found to inhibit mitochondrial respiration in a dose-dependent manner, as well as via interaction with mitochondrial toxins. PD patient studies have provided evidence for a 35% decrease in complex I activity, suggesting that dopamine may be a potential contributor to OS and mitochondrial dysfunction within dopaminergic neurons, leading to neuronal damage and degeneration. However, without stress conditions, PINK1 KO mice neurons also show reduced ATP levels, and isolated mitochondria from PINK1 KO cerebral tissue demonstrate a significant and progressive reduction in ΔΨm, although this may be associated with age.

Mitochondrial abnormalities in the dopaminergic neurons of PD patients and dopamine neuronal cultures were investigated by Chung et al. (2016), who identified mitochondrial defects and protein accumulation in parkin and PINK1 induced pluripotent stem cell (iPSC)-derived midbrain neuronal populations [58]. These neuronal populations revealed a considerable accumulation of abnormal mitochondria, in comparison with control iPSCs, which comprised of a smaller fraction of abnormal mitochondria after 75 days of differentiation. The abnormal mitochondria were also observed as being larger in size compared with the control neurons. This suggests that mutations in parkin and PINK1 are capable of triggering alterations in mitochondrial homeostasis, initiating multiple cellular pathogenic modifications. Another study with parkin mutant iPSC neurons showed increased OS and reduced GSH levels, accompanied by Nrf2 pathway activation [69]. Increases in OS were supported in post-mortem PD brains where increased levels of 4-hydroxyl-2-nonenal (HNE), a by-product of lipid peroxidation, and 8-hydroxy-guanosine and 8-hydroxy-deoxyguanosine, by-products of DNA and RNA oxidation, respectively, were identified [70]. Post-mortem PD brains were also found to have an increased expression of Nrf2 pathway proteins, including Nrf2 and NQO1, suggesting that the Nrf2 pathway is activated in iPSC-derived neurons in order to protect neurons from further oxidative damage. The Nrf2-ARE pathway is often downregulated in several neurodegenerative disorders, suggesting that parkin mutations are capable of impairing the Nrf2 antioxidant pathway [38]. Nevertheless, the cell is able to compensate by upregulating antioxidant protective pathways, and therefore provides an important therapeutic target. 

##### α-Synuclein and Mitochondrial Dysfunction

α-synuclein aggregation and accumulation is strongly associated with several neurodegenerative diseases, including PD, dementia with Lewy bodies (DLB), and multiple system atrophy (MAS). Evidence of α-synuclein aggregates have been recognised in post-mortem PD brains to accumulate within mitochondria, despite its predominant localisation to the cytosol [71,72]. Overactivation of autophagy is a potential link between mitochondrial dysfunction and α-synuclein accumulation [73], and intracellular accumulation has been shown to be capable of inducing OS and increasing ROS levels, as well as inducing mitochondrial fragmentation [74]. Although the mechanisms are not yet fully understood, evidence has shown that α-synuclein has direct effects on mitochondrial morphology due to mutations encoding α-synuclein increasing mitochondrial fragmentation as well as affecting mitochondrial biogenesis via the regulation of PGC1α [38,75]. A study using human dopaminergic neurons with overexpressed mutant A53T α-synuclein has provided evidence that basal and toxin-induced oxidative/nitrosative stress results in an inhibition of the MEF2C-PGC1α transcriptional network [75]. Myocyte enhancer factor 2 (MEF2C) is a transcription factor that binds to the PGC1α promoter activating it, and evidence has shown that the inhibition of this network contributes to mitochondrial dysfunction and apoptotic cell death in PD [75,76]. Moreover, this toxic protein has been found to interact with mitochondrial membranes, including in neuronal synapses, and has been associated with impaired complex I-dependent respiration and reduced ΔΨm, therefore impacting energy production and mitochondrial ROS production [37]. Recent evidence has identified an interaction between α-synuclein and MRC complex I, resulting in reduced mitochondrial activity and exacerbated ROS production [77]. The relevant molecular target has been found to be the *N*-terminal domain of α-synuclein, which targets the aggregated protein towards MRC complex I. It has also been reported that soluble α-synuclein is capable of directly interacting with mitochondria-associated endoplasmic reticulum membranes (MAM), which can cause an overexpression of the proteins that alter mitochondrial morphology and cause mitochondrial fragmentation in cell models of PD [78]. Additionally, α-synuclein has been found to bind to OMM proteins, including the voltage-dependent anion-selective channel 1 (VDAC1), which has been implicated as a factor contributing to mitochondrial dysfunction in sporadic PD [35]. Levels of VDAC1 in nigral neurons of patients with sporadic PD were found to be reduced as a consequence of α-synuclein aggregation. 

Thereby, parkin, PINK1, and α-synuclein represent important therapeutic targets in order to alleviate mitochondrial dysfunction and bioenergetic deficits, leading to neurodegeneration in both sporadic and familial PD.

### 3.3. Inherited Metabolic Disorders

Inherited metabolic disorders (IMDs) are a large group of single gene disorders affecting several features of cellular metabolism [79]. These disorders are traditionally well-known as enzymopathies due to disturbances in specific pathways leading to the accumulation of toxic metabolites or intermediate products and/or reduced synthesis of an essential metabolite. The majority of IMDs involve the CNS, resulting in major neurodevelopmental disruptions during the first stages of life, as a consequence of the impairment in cellular function due to the presence of toxic metabolites and intermediates that accumulate in these disorders [80]. In the brain, these metabolites can behave as neurotransmitters or stimulate biological pathways, which can result in neuronal dysfunction and ultimately neurodegeneration. Several studies from patients and animal models have provided evidence to demonstrate that metabolic alterations in mitochondria and energy dysfunction may play a vital role in the pathophysiology of neurological disease in IMDs, and that bioenergetic disruption is a crucial biochemical event leading to neurodegeneration in metabolic disease [81]. 

#### 3.3.1. Methylmalonic Acidaemia

**Figure 6:**
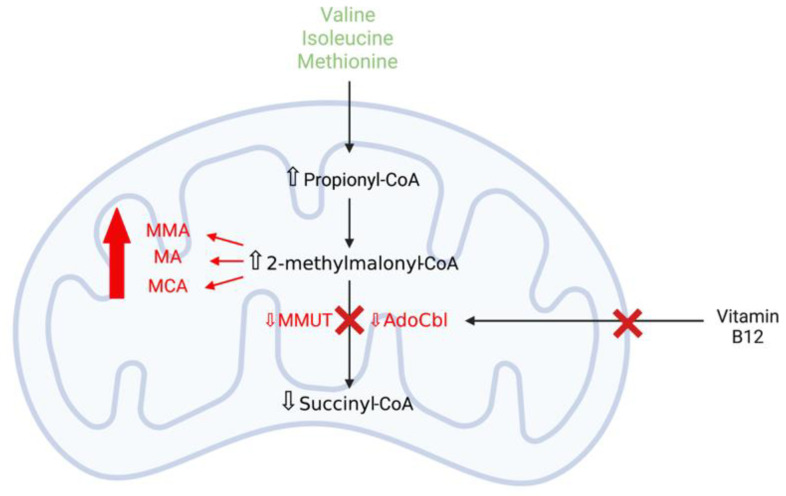
Defective metabolic pathway in methylmalonic acidaemia. A deficiency in the activity of MMUT or defects in the uptake, transport, and synthesis of the MMUT cofactor, AdoCbl, causes methylmalonic acidaemia. Vitamin B12 is an essential cofactor for MMUT. MMUT is essential for the degradation of the amino acids, valine, isoleucine, and methionine, and defects in this enzyme increase the concentration of MMA and its secondary metabolites, MA and MCA, in body fluids. These metabolites are mitochondrial toxins capable of inducing oxidative stress and excitotoxicity (created using biorender.com). MMA—methylmalonic acid; MA—malonic acid; MCA—2-methylcitrate; MMUT—l-methylmalonyl-CoA mutase; AdoCbl—5′-deoxyadenosylcobalamin; CoA—coenzyme A.

Methylmalonic acidaemia (MMAemia), the most common organic acidaemia, is an autosomal recessive IMD and is a severe, multi-system disease of abnormal organic acid metabolism [82]. MMAemia is caused by deficiency of the mitochondrial enzyme l-methylmalonyl-CoA mutase (MMUT) activity, which is essential for the degradation of amino acids such as valine, isoleucine, and methionine and lipids, or by defects in the uptake, transport, or synthesis of the MMUT cofactor, 5′-deoxyadenosylcobalamin (AdoCbl) (Figure 6) [83,84]. Defects in the activity of MMUT increase the concentration of methylmalonic acid (MMA), the primary metabolite accumulating in MMAemia, in patient tissues and body fluids, including urine and cerebrospinal fluid (CSF). Additionally, the accumulation of secondary metabolites, including 2-methylcitrate (MCA), malonic acid (MA), propionate, and 3-hydroxypropionate, are also detected and are mitochondrial toxins capable of inducing excitotoxicity and OS [84,85]. MMAemia has a wide clinical spectrum, with the disease manifestations ranging from the fatal neonatal period to adulthood. The severe, early-onset form of the disease typically presents in neonates with lactic acidosis, hyperammonaemia, lethargy, failure to thrive, and neurological defects, including developmental delay, hypotonia, convulsions, metabolic stroke (acute and chronic basal ganglia involvement), and coma [82]. Long-term complications, such as renal failure and neurological diseases, are frequently observed in MMAemia patients. 

##### Mitochondrial Dysfunction and Oxidative Stress Contribute to Neurodegeneration in Methylmalonic Acidaemia

**Figure 7:**
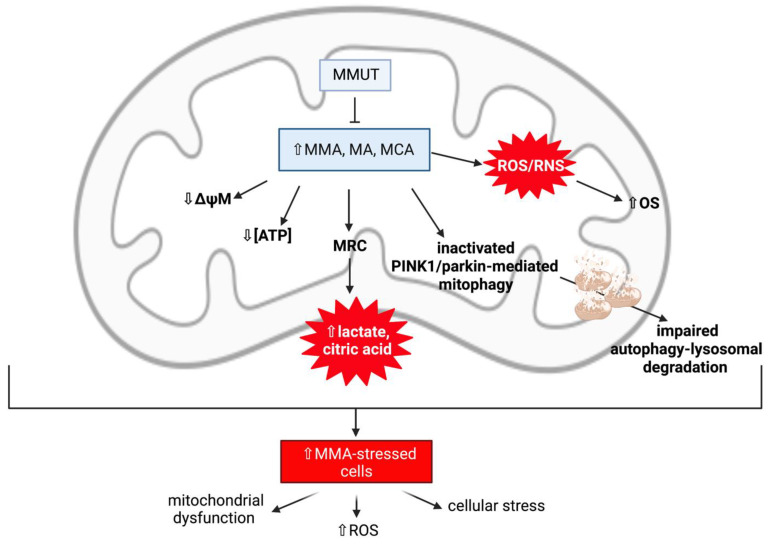
Mechanisms resulting in mitochondrial dysfunction and the development of neurodegeneration due to deficient L-methylmalonyl-CoA mutase activity and the accumulation of methylmalonic acid and secondary metabolites in methylmalonic acidaemia. Mitochondrial accumulation of these toxic organic acids leads to consequences that affect the mitochondrial structure and function such as reduced ΔΨm and ATP; impaired MRC function resulting in increases in lactate and citric acid; inactivated PINK1/parkin-mediated mitophagy, resulting in accumulation of damaged mitochondria that are not targeted for removal via the autophagy−lysosomal system; and increases in reactive oxygen/nitrogen species causing increased oxidative stress. A combination of these factors results in elevations in the number of methylmalonic acid-stressed cells, leading to mitochondrial dysfunction and cellular stress, which are factors responsible for the development of neurodegeneration in methylmalonic acidaemia (created using biorender.com). ΔΨm—mitochondrial membrane potential; ATP—adenosine triphosphate; MRC—mitochondrial respiratory chain; PINK1—PTEN-induced kinase 1; MMUT—L-methylmalonyl-CoA mutase; MMA—methylmalonic acid; MA—malonic acid; MCA—2-methylcitrate; ROS/RNS—reactive oxygen/nitrogen species; OS—oxidative stress.

Several animal MMAemia models and patient studies have demonstrated that mitochondrial dysfunction is an important contributor to the development of neurodegeneration in MMAemia [81]. In patient studies, this has been supported by elevated levels of lactic acid—an indicator of impaired MRC function—in the CSF and globus pallidus of the brain, as well as elevated levels of the TCA cycle intermediate, citric acid, in the blood (Figure 7). These elevated biomarkers are suggestive of an impairment of mitochondrial metabolism, such as mitochondrial abnormalities and MRC enzyme complex inhibition, ultimately leading to neuronal loss. In a study undertaken by Brusque et al. (2002), millimolar MMA concentrations consistent with those reported in MMAemia patients were found to inhibit the MRC in the cerebral cortex of young rats [85]. The MRC complex I activity was significantly inhibited by the presence of MMA, which was found to be a weak inhibitor of complex II (succinate dehydrogenase) activity, suggesting that MMA may be considered as a neurotoxin. Interestingly, inhibition of complex I activity has also been observed in the basal ganglia of PD patients. Therefore, it may be assumed that inhibition of the MRC complexes by MMA is perhaps very destructive to the brain, as OXPHOS is a highly active system in the cerebral tissue [85,86]. 

Other studies have suggested that MMA may induce secondary excitotoxicity and OS, which are well-established mechanisms involved in neuronal damage [87]. Experimental evidence has found several OXPHOS defects in MMAemia patient and MMUT KO mice models, resulting in perturbations in antioxidant defence systems, which, as a consequence, cause oxidative damage, leading to MMA-induced deficient energy metabolism. Studies with MMAemia-patient fibroblasts have identified an increased expression of multiple proteins related to OS and apoptosis, including cytochrome *c* and MnSOD, as well as significant increases in intracellular ROS levels [85]. Moreover, Atkuri et al. (2009) found that MMAemia patients expressed lower levels of GSH in multiple immune cells compared with healthy controls [88]. In cultured neurons, MMA accumulation was also found to reduce the ATP/ADP ratio and cause mitochondrial membrane depolarisation and ion gradient failure, leading to necrotic and apoptotic cell death [89,90,91]. Additionally, secondary metabolites of MMA, such as MCA, MA, and propionyl-CoA, have been demonstrated to also inhibit the activity of the MRC and TCA cycle [87]. It has been suggested that neuronal damage in MMAemia is not caused by MMA alone, but by the synergistic contribution of alternate propionyl-CoA oxidation metabolites. These secondary metabolites are considered as neurotoxic agents, as they have been demonstrated to be capable of impairing OXPHOS via pyruvate dehydrogenase complex inhibition and inducing hyperammonaemia via *N*-acetylglutamate synthetase inhibition. 

##### MMUT Deficiency and Mitochondrial Dysfunction 

Although the mechanisms are not yet fully understood, recent evidence has demonstrated that a deficiency in the gene encoding methylmalonyl-CoA mutase, *MUT*, affects the efficient functioning of the PINK1/parkin pathway in order to remove dysfunctional mitochondria via the autophagy-lysosomal system, a degradation system required for the removal of harmful cytosolic entities such as misfolded proteins, aged, and/or damaged organelles [20,81,92]. This suggests that there may be a primary link between MMUT deficiency, mitochondrial dysfunction, and cellular stress in the development of neurodegeneration in MMAemia.

Inactivating mutations in the *MUT* gene can result in a loss-of-function of the MMUT enzyme activity, and these mutations can either be a complete (*MUT^0^*) or partial (*MUT^-^*) loss-of-function, leading to the accumulation of toxic organic acids within the mitochondrial matrix, initiating mitochondrial network structural and functional abnormalities. MMUT is expressed in various tissues, including the basal ganglia of the brain [93], however it is highly expressed within the mitochondria of kidney tubular cells, therefore the consequences of MMUT deficiency on mitochondrial network function and homeostasis have been investigated using these cells [92]. In MMAemia-patient kidney tubular cells, an MMUT deficiency can result in the accumulation of damaged and dysfunctional mitochondria as a consequence of deficient MMUT inactivating PINK1/parkin-mediated mitophagy, therefore producing excessive levels of ROS and causing cellular distress [20]. In this same study (Luciani et al., 2020), a link between MMUT deficiency and autophagy induction was reported, suggesting that an MMUT deficiency may impair mitochondrial homeostasis and function via compromised PINK1/parkin-directed priming of MMA-stressed mitochondria to autophagic-lysosomal degradation [20]. Both MMA and control cells were treated with the mitochondrial complex I inhibitor [94], rotenone, in order to induce mitochondrial damage. This study observed, under normal and stress-induced conditions, a reduced amount of parkin clusters and reduced translocation of parkin to damaged mitochondria in MMA cells, in addition to mitochondrial abnormalities such as mitochondrial fragmentation, reduced ΔΨm, and impaired mitochondrial respiration and ATP generation [20]. As a consequence of the defective PINK1/parkin priming mechanisms, mutant *MUT* cells fail to transport their damaged mitochondria to the autophagy-lysosomal degradation systems, therefore triggering an accumulation of MMA-diseased mitochondria within the cell [90]. Thus, the disrupted clearance of MMA-stressed mitochondria triggers a level of mitochondrial dysfunction that causes cellular distress and damage. 

In order to demonstrate the central role of MMUT in maintaining mitochondrial network function and homeostasis, Chen et al. (2020) analysed a KO *MUT* zebrafish model that displayed an abnormal mitochondrial morphology characterised by perturbed mitochondrial cristae organisation, in addition to impaired mitochondrial bioenergetics, contributing to increased mitochondrial OS [92]. This was also observed in an earlier experimental study on rats, where chronic exposure to MMA resulted in mitochondrial swelling and disorganised cristae of the tubulum epithelium [95]. Moreover, studies with mutant kidney cells have demonstrated that mitochondrial homeostasis and function can be improved by therapeutic interventions that target the PINK1/parkin pathway by activating mitophagy-mediated degradation in order to avoid mitochondrial-derived cellular stress and damage, as well as maintaining efficient mitochondrial quality control mechanisms [20].

#### 3.3.2. Lysosomal Storage Disorders

Lysosomal storage disorders (LSDs) are a heterogenous group of rare IMDs caused by genetic mutations that cause absence or loss-of-function of lysosomal hydrolases and/or non-lysosomal protein transporters, leading to the progressive accumulation of undigested material within the lysosomal and/or autophagic systems, therefore affecting lysosomal function and reducing cell survival [96]. Most LSDs display an autosomal recessive mode of inheritance, for example Gaucher Disease (GD) and Niemann Pick Type C (NPC), although some LSDs are inherited in an X-linked pattern, for example Mucopolysaccharidosis Type II (MPSII) [97,98]. The clinical manifestations of LSDs vary depending on age of onset, severity, and the specific substrate and site of accumulation, and are multisystemic diseases characterised by hepatic, renal, cardiovascular, and pulmonary and neurological manifestations [98,99,100]. The majority of LSDs have a profound influence on the CNS and frequently present with neurological symptoms leading to neurodegeneration in multiple brain regions, including the cerebellum, cortex, and hippocampus [99]. Moreover, the accumulation of undigested material within the lysosomes also affects the function of other organelles, including the mitochondria, leading to secondary consequences such as impaired autophagy, mitochondrial dysfunction, inflammation, and apoptosis. 

##### Mitochondrial Dysfunction in Lysosomal Storage Disorders

**Figure 8:**
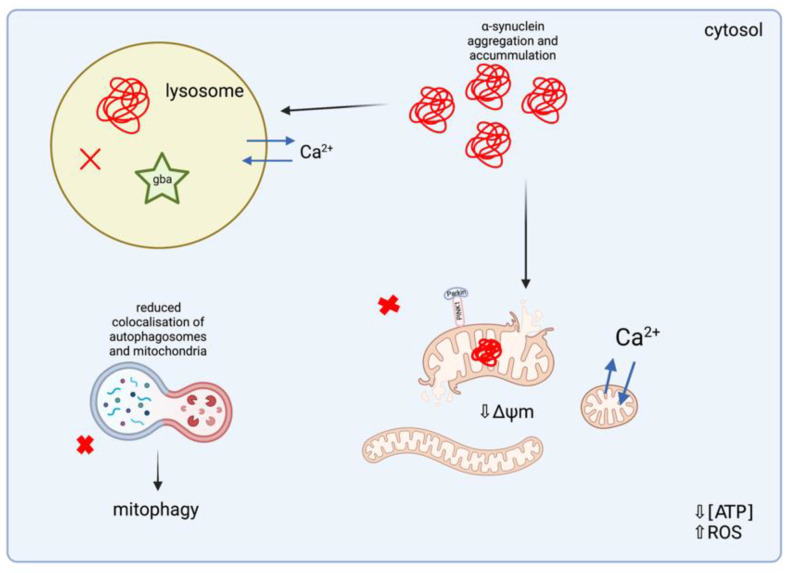
Molecular mechanisms leading to mitochondrial dysfunction in lysosomal storage disorders. Lysosomal dysfunction is associated with dysregulated autophagy/mitophagy pathways, leading to the accumulation of dysfunctional mitochondria within the cell. Mitochondria can be further damaged by the accumulation of aggregated α-synuclein causing altered mitochondrial morphology (small, rounded fragmented and elongated mitochondria). α-synuclein accumulation is also associated with dysfunctional mitochondrial bioenergetics, including reduced ΔΨm and ATP, increases in ROS levels, and altered Ca^2+^ homeostasis, which are events leading to cell death. Impaired mitophagy also occurs as a consequence of impaired PINK1/parkin recruitment and/or function. Reduced recycling and the removal of mitochondria by mitophagy increases the number of damaged mitochondria within the cell due to reduced colocalisation of autophagosomes and mitochondria (created using biorender.com). ΔΨm—mitochondrial membrane potential; ATP—adenosine triphosphate; ROS—reactive oxygen species; PINK1—PTEN-induced kinase 1; gba—glucocerebrosidase.

Impaired mitochondrial function has been suggested as a collective pathogenic feature of LSDs, as the majority of LSDs frequently present with primary lysosomal impairment in addition to mitochondrial dysfunction [101]. Experimental studies have reported mitochondrial morphological abnormalities, such as mitochondrial fragmentation and/or elongation, as well as dysfunctional mitochondrial bioenergetics, as a consequence of impaired mitochondrial respiration, reduced MRC activity, and/or decreased ΔΨm (Figure 8). In mouse models of neuropathic GD, the most common LSD caused by mutations in the *glucocerebrosidase* (*gba*) gene, which encodes for the lysosomal enzyme, glucocerebrosidase (GCase), neurons, and astrocytes that do not express *gba,* were found to have defective autophagic and proteasomal machinery, causing a primary lysosomal defect and leading to an accumulation of dysfunctional mitochondria within the cell [102]. Furthermore, Osellame et al. (2013) reported evidence of impaired mitochondrial function in neurons and astrocytes of type II GD, as morphological abnormalities were indicated by small and fragmented mitochondria [102]. The resting ΔΨm was measured in *gba* KO (*gba^−/−^*) mice models and was found to be significantly lower in comparison with mice neurons and astrocytes derived from *gba* homozygous (*gba^+/+^*) and *gba* heterozygous (*gba^+/−^*) mutations. *gba^+/+^* and *gba^−/−^* neurons were incubated with oligomycin—an ATPase inhibitor —in order to assess the function of the MRC, demonstrating that the ΔΨm in *gba^+/+^* neurons was not affected by incubation with oligomycin, but was significantly reduced in *gba^−/−^* neurons, suggesting that the reduced ΔΨm is maintained by ATP synthase (complex V) working in reverse, which is a mechanism implicated in PINK1 KO-associated PD. Moreover, confocal imaging of neurons and astrocytes located within the midbran indicated significant mitochondrial fragmentation in *gba^−/−^* cells, in comparison with *gba^+/+^* and *gba^+/−^* cells, suggesting that GD can cause increased mitochondrial fragmentation and MRC impairments, including reduced complex I and complex II + III activity, leading to mitochondrial dysfunction. Interestingly, evidence has also demonstrated an association between an absence or loss-of-function of *gba* and an increased risk of PD in GD patients, as the genetic causes of familial PD are thought to induce the same biochemical and molecular events that underlie the pathogenesis of several LSDs. 

Moreover, a study carried out by Cleeter et al. (2013) identified a significant reduction in ATP synthesis involving both MRC complex I and complex II/III-linked ADP phosphorylation, as a result of a 20-day incubation with a selective inhibitor of GCase activity, conduritol-β-epoxide (CβE), with inhibited levels of GCase activity consistent with those reported in GD patients [103]. Despite an observed significant reduction in ATP synthesis, individual MRC protein activities were unaltered, suggesting that defective ATP synthesis may be due to dysfunctional electron transfer in the MRC and/or defective mitochondrial membrane. Inhibition of GCase with CβE caused mitochondrial function abnormalities and increased OS, which are defects frequently observed in the PD brain and mitochondrial disease [22,103]. Additionally, following CβE treatment, a significant and progressive elevation in free radical generation was observed, which may have contributed to the observed mitochondrial fragmentation as a result of increased OS [103]. 

##### Oxidative Stress in Lysosomal Storage Disorders

Evidence of elevated OS has been reported in patient GD fibroblasts through the presence of an increased generation of superoxide ions from non-phagocytic NADPH oxidase together with protein carbonyls from augmented protein oxidation [104]. It is possible that OS may also result from an accumulation of damaged mitochondria whose aberrant MRC function may become a major source of ROS generation, contributing to OS-induced cellular damage [101]. Furthermore, evidence of OS has also been reported in NPC human fibroblasts; post-mortem cerebellum; and MPS IV patient urine, plasma, and leukocytes, where increased protein oxidation, lipid peroxidation, and DNA damage have been described [99]. In NPC human fibroblasts, a reduced SOD2 and/or CAT activity was observed, as well as diminished GSH levels, in addition to MPS IV patient erythrocytes showing decreased GSH activity, suggesting the presence of increased OS in other LSDs. Furthermore, mitochondrial-associated OS events are also known to be dysfunctional in the PD brain, which may result from a loss of cerebral GSH status, which has been reported in post-mortem studies [35].

##### Dysregulated Mitochondrial Biogenesis and Mitophagy in Lysosomal Storage Disorders 

The accumulation of defective mitochondria may possibly be due to dysregulated mitochondrial biogenesis and mitophagy pathways, which have been reported in animal and cellular models of several LSDs [105]. Impaired mitophagy through impaired PINK1/parkin recruitment and/or function has recently been investigated in mouse models of GD, which have shown reduced colocalisation of autophagosomes and mitochondria, therefore leading to reduced recycling of damaged mitochondria in mice primary neurons [99]. This was demonstrated in a study undertaken by Osellame et al. (2013), where *gba^-/-^* neurons failed to recruit parkin despite a reduction in ΔΨm. In order to assess the failed recruitment of parkin, cells were treated with carbonyl cyanide 4-(trifluoromethoxy)phenylhydrazone (FCCP)—an uncoupler of mitochondrial OXPHOS leading to the collapse of ΔΨm—in order to promote PINK1 accumulation at the OMM [102]. However, once the mitochondria were depolarised, the *gba^-/-^* neurons showed no difference in the levels of PINK1 in comparison to the controls, confirming that a reduced ΔΨm in *gba^-/-^* neurons is not sufficiently low enough for parkin recruitment, suggesting that ΔΨm must be near to complete dissipation in order to be selected for turnover. As a consequence, damaged mitochondria remain unmarked for turnover via mitophagy, resulting in impaired and defective mitochondria accumulating in *gba^-/-^* neurons, therefore compromising neuronal cell function. In another study by Moren et al. (2019), GD cellular models derived from neural crest stem cells containing *gba^+/+^* and *gba^+/−^* mutations were found to increase PGC1α levels under carbonyl cyanide-m-chlorophenyl-hydrazine (CCCP)-induced mitophagy—another mitochondrial OXPHOS uncoupler promoting cell death [106]—therefore increasing the mitochondrial content via the activation of mitochondrial biogenesis [107]. As a result of treatment with CCCP, mitochondrial depolarisation led to a significant elevation in PGC1α in *gba^+/+^* cell lines in comparison with control and *gba^+/−^* cell lines, which is possibly a compensatory mechanism for the underlying mitochondrial defects and GCase deficiency in GD. This study identified that impaired turnover of depolarised mitochondria and GCase-deficient neurons in GD are possible mechanisms that may be associated with early mitochondrial impairments and dysfunction in this disorder. 

##### Mitochondrial Dysfunction and α-Synuclein Aggregation in Lysosomal Storage Disorders

The accumulation of protein aggregates, such as α-synuclein, may further deteriorate mitochondrial function and have been reported in post-mortem cerebral samples of LSD patients [99]. Although α-synuclein accumulation is strongly associated with both sporadic and familial forms of PD, the assessment of numerous LSD patient samples has revealed the presence of Lewy bodies and neurotoxic intracellular accumulation of α-synuclein in cerebral tissue, including in the cortex, hippocampus, and substantia nigra [99,108]. Interestingly, Lewy bodies containing aggregated α-synuclein have been associated with mitochondrial fragmentation and dysfunction in several cellular models of LSDs [99]. This was investigated by Choi et al. (2012), where brain homogenates from the cerebral cortex of the post-mortem brain samples of patients with *gba* mutations were found to contain aggregated α-synuclein [109]. This study analysed the presence of intracellular α-synuclein inclusions using SDS-PAGE and Western blot, demonstrating that most patients with *gba* mutations exhibited bands of insoluble oligomeric forms of α-synuclein, in comparison with the controls and GD patients without *gba* mutations. Cerebral tissue from animal models with neuronal variants of GD revealed disrupted mitochondrial cristae and the presence of rounded, fragmented mitochondria associated with aggregated α-synuclein accumulation, as well as reduced oxygen consumption and reduced ATP levels [108]. These protein aggregates have been found to colocalise with mitochondria, suggesting that α-synuclein may cause a direct impairment to mitochondrial function, although it has not yet been fully elucidated whether α-synuclein accumulation is a primary cause of mitochondrial damage in LSDs or whether it is a secondary consequence caused by increased generation of ROS and/or impaired autophagic systems [99]. 

In a study undertaken by Luth et al. (2014), the accumulation of α-synuclein was demonstrated to directly inhibit MRC complex I activity, as well as reduce ΔΨm and increasing ROS generation and alter calcium homeostasis [110]. Therefore, these studies suggest that the accumulation of protein aggregates, including α-synuclein, are risk factors for the development of mitochondrial dysfunction in LSDs. Moreover, the presence of Lewy bodies has also been reported in other LSDs, including the presence of phosphorylated α-synuclein in MPS III patient cerebral tissue samples, in addition to samples of cerebral tissue from NPC patients, which were positive for the presence of aggregated α-synuclein [111,112]. The plasma of GD and NPC patients, in addition to NPC lymphoblasts, were also reported to contain α-synuclein oligomers [113,114]. LSDs are complex disorders, therefore therapeutic strategies that target several different mitochondrial-associated molecular pathways may be beneficial for improving mitochondrial function and quality control mechanisms, particularly in neuronal cells, in order to alleviate mitochondrial dysfunction and neurodegeneration in LSDs.

## 4. Therapeutic Approaches

### 4.1. Targeting Dysfunctional Mitochondria to Induce Mitochondrial Biogenesis 

Inherited and non-communicable diseases that result in CNS disturbances are often associated with mitochondrial dysfunction, therefore therapeutics that are capable of targeting mitochondria in order to prevent mitochondrial-associated development of neurodegeneration may be beneficial in the treatment of these disorders. Several experimental studies have identified potential therapeutic compounds that have the capacity to induce mitochondrial biogenesis or the generation of new, functional mitochondria within cells in order to stimulate cellular repair and regeneration [115]. Mitochondrial biogenesis is a process that is defined as the growth and division of pre-existing mitochondria, and is triggered by numerous environmental stressors, including OS [116]. PGC1α is the most inducible and responsive member of the PGC-1 family, and has been shown to drive mitochondrial biogenesis in response to various environmental signals [117]. PGC1α can be directly activated via silent mating type information regulation 2 homolog (SIRT1)-mediated deacetylation, resulting in increased co-activation of SIRT1-targeted transcription factors, which is an important cellular mechanism in order to increase mitochondrial metabolism in response to stress conditions [118]. Additionally, the phosphorylation of kinases, including AMP-dependent kinase (AMPK), can directly regulate PGC1α activity. However, AMPK is also capable of regulating PGC1α activity indirectly, by increasing NAD^+^ via increased fatty acid oxidation, therefore enhancing SIRT1 activity and inducing SIRT1-mediated deacetylation. The activation of mitochondrial biogenesis can be achieved via the pharmacological induction of PGC1α, which has been demonstrated to improve oxidative metabolism and mitochondrial bioenergetics, potentially providing neuroprotective effects and improving overall cellular function [115,117,119]. Therefore, mitochondrial biogenesis is an interesting novel therapeutic approach in order to target mitochondrial dysfunction in diseases that involve neurodegeneration.

#### 4.1.1. Natural Polyphenols

##### Resveratrol

Natural polyphenols, including resveratrol (3,5,4′-trihydroxystilbene) and curcumin (1,7-bis(4-hydroxy-3-methoxyphenyl)-1,6-heptadiene-3,5-dione), are produced in plants in response to injury and stress, and are found in several food sources [120]. These redox-active compounds have become an interesting mitochondria-targeting therapeutic in order to preserve the structure and function of the mitochondria and neurons [121]. Polyphenols exhibit antioxidant and neuroprotective properties including the ability to activate transcription factors such as Nrf2, which are involved in the expression of antioxidant enzymes, and can directly regulate the mitochondrial apoptosis system and induce mitochondrial biogenesis in order to improve mitochondrial mass and function [121,122,123]. These compounds have shown many beneficial effects in the mitochondria, however they often exhibit very low bioavailability, poor absorption, and rapid metabolism, notably in the brain [120,124], which may compromise the effectiveness of these compounds in neurometabolic and neurodegenerative diseases. 

**Figure 9:**
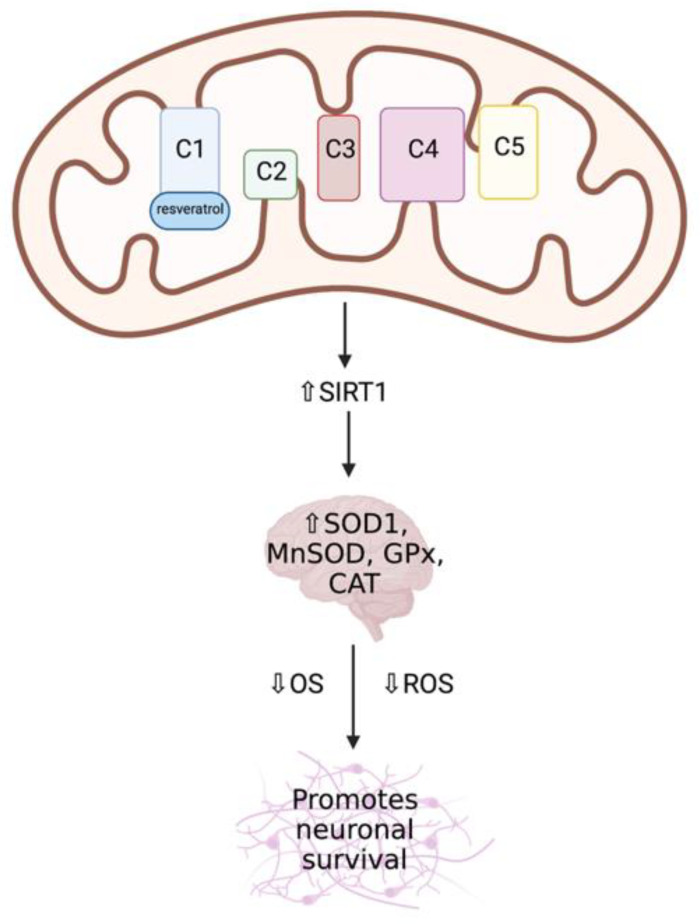
Mitochondrial respiratory chain complex I is a direct target of resveratrol. The interaction between mitochondrial respiratory chain complex I and resveratrol results in the simulation of SIRT1, leading to SIRT1-dependent upregulation of several antioxidants in the brain, including SOD1, MnSOD, GPx, and CAT. Resveratrol treatment promotes neuronal survival in PD (created using biorender.com). C1–C5—MRC complexes 1–5; SIRT1—silent mating type information regulation 2 homolog; SOD1—superoxide dismutase 1; MnSOD—manganese superoxide dismutase; GPx—glutathione peroxidase; CAT—catalase; OS—oxidative stress; ROS—reactive oxygen species; PD—Parkinson’s disease.

Several experimental studies have identified mitochondria as the key targets of resveratrol, as it has been shown to be a regulator of MRC function, capable of inducing mitochondrial biogenesis and reducing ROS production via its interaction with SIRT1 and AMPK via PGC1α [116,125]. Gueguen et al. (2015) identified MRC complex I as a direct target of resveratrol action (Figure 9), resulting in an increase in its enzymatic activity following treatment with low doses of the compound (1–5 μM) [125]. This interaction between MRC complex I and resveratrol results in the stimulation of key signalling pathways, including the SIRT1-dependent upregulation of several antioxidant enzymes in the brain, such as SOD1 and SOD2, GPx, and CAT [125,126,127]. Resveratrol has been found to promote neuronal survival via inducing apoptotic cell death, in addition to the regulation of secondary consequences of disease pathogenesis, including inhibiting OS and preventing neuro-inflammation [120,128]. In primary fibroblast cultures from patients with early-onset PD, resveratrol treatment was found to induce apoptosis in order to efficiently remove damaged mitochondria and misfolded proteins [129]. Increased mRNA expression of several PGC1α target genes was also identified in parkin-mutated fibroblasts following resveratrol treatment, which was then demonstrated to improve mitochondrial oxidative function, while simultaneously reducing OS and increasing mitochondrial biogenesis [130]. Interestingly, resveratrol treatment resulted in increased MRC complex I and citrate synthase (CS) activity—a biomarker for mitochondrial enrichment—increased ATP production and reduced lactate concentrations, suggesting an induction of mitochondrial biogenesis. Resveratrol is able to rapidly cross the BBB, however it should be noted that resveratrol is rapidly metabolised and has both a low solubility and bioavailability, which may reduce the efficacy of this potential therapeutic compound, although this may be improved with the addition of bioenhancers or drug-delivery carriers [120,131]. In vitro animal studies with bioenhancers have shown increased plasma concentrations of resveratrol through the use of combination therapies, for example, combining resveratrol and hydroxypropyl-β-cyclodextrin, which increased the solubility and absorption of this compound, in addition to nanoformulations of resveratrol, which increased its surface area for absorption [120]. However, the effects of bioenhancers have not yet been widely studied in humans.

##### Curcumin

Curcumin has a wide range of health promoting functions, including cytoprotective and neuroprotective properties, in addition to its favorable absorption, bioavailability, and long half-life in the CNS [132]. The long half-life of curcumin in the CNS (6–7 h in plasma) is thought to be due to its amphiphilic nature and a high concentration of lipids within the brain, meaning that this compound may be beneficial in patients who present neurologically [132,133]. Several experimental studies have suggested that curcumin can interact simultaneously with multiple molecular targets, resulting in a cascade of biochemical and molecular events and activating multiple signalling molecules, such as AMPK, SIRT1, and Nrf2, and therefore protecting the cells from oxidative injury by increasing mitochondrial mass and protein expression elicited by the induced mitochondrial biogenesis [119,120,134]. Curcumin has also been shown to exhibit a protective effect against mitochondrial dysfunction and apoptosis, in addition to improving oxygen consumption rates, increasing cell viability, and ΔΨm in PINK1-deficient cells [135]. Models of PD have also demonstrated the potential neuroprotective effects of curcumin, as rat SNpc neurons were shown to be protected from apoptosis following curcumin treatment, leading to increased dopamine levels within the striatum, and reduced cytotoxicity and cell viability [135,136,137]. Curcumin has been shown to prevent cytochrome c release and inhibit α-synuclein-induced neuronal toxicity and death in a PC12 cell model of PD [138,139,140]. Moreover, curcumin treatment was demonstrated to be beneficial in an NPC1 mice model, where triple combination therapy involving curcumin, miglustat (targets sphingolipid synthesis and storage in GD and NPC), and ibuprofen (reduces CNS inflammation) was found to enhance neuroprotection and cell survival, in addition to ameliorating the autophagic flux function [141]. 

#### 4.1.2. Bioactive Quinones

##### Coenzyme Q_10_ (CoQ_10_)

**Figure 10:**
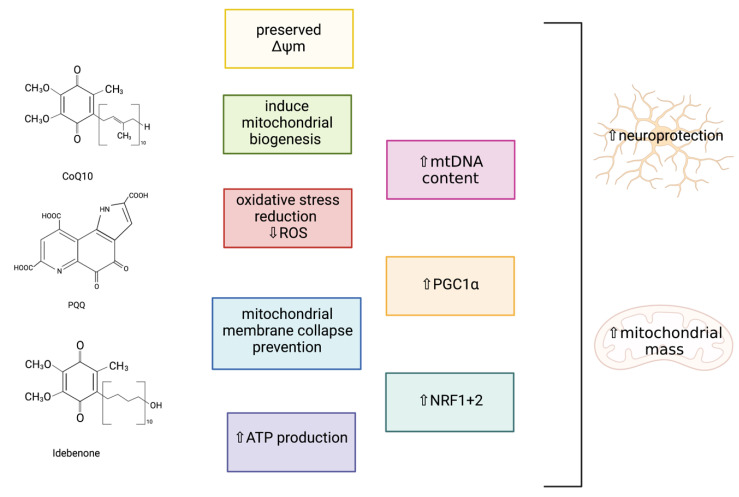
Summary of the neuroprotective effects of the following bioactive quinones: coenzyme Q10, pyrroloquinoline quinone, and idebenone. These compounds have been reported in the literature as having roles as neuroprotective agents, in addition to upregulating the mitochondrial mass and function in neurodegeneration (created using biorender.com). CoQ10—coenzyme Q10; PQQ—pyrroloquinoline quinone; ΔΨm—mitochondrial membrane potential; ROS—reactive oxygen species; ATP—adenosine triphosphate; mtDNA—mitochondrial DNA; PGC1α—peroxisome proliferator-activated receptor gamma coactivator 1-alpha; NRF1 + 2—nuclear respiratory factor 1 + 2.

CoQ_10_ is a potent antioxidant and essential cofactor carrying electrons from complexes I and II to complex III of the MRC, and is a well-known activator of PGC1α [142,143]. CoQ_10_ has been suggested to have a role as a neuroprotective agent, as this lipophilic antioxidant has been shown to improve cognitive function, facilitate ATP synthesis, and upregulate mitochondrial function in neurodegeneration [144]. A study carried out by Somayajulu et al. (2005) investigated the role of CoQ_10_ as a neuroprotective agent when neuronal cells were exposed to hydrogen peroxide (H_2_O_2_)-induced OS [145]. In human neuroblastoma (SH-SY5Y) cells pre-treated with water-soluble CoQ_10_ following H_2_O_2_ treatment, a significant reduction in mitochondrial ROS generation, preserved ΔΨm, increased mitochondrial ATP production, and the prevention of mitochondrial membrane collapse were observed in comparison with the control mitochondria. This suggests that CoQ_10_ can act as a potent antioxidant at the mitochondrial level, as CoQ_10_ has been demonstrated to stabilise the mitochondrial membrane and increase mitochondrial numbers as a consequence of H_2_O_2_-induced OS, which may be due to the ability of CoQ_10_ to increase PGC1α signalling [142,145,146]. Studies with senescence-accelerated mouse (SAM) models have revealed that a diet supplemented with CoQ_10_ can induce pathways involving SIRT1, SIRT3, and PGC1α, preventing mitochondrial deterioration by increasing OS resistance [143]. In this study, Western blot analyses demonstrated that CoQ_10_ induced the deacetylation and activation of PGC1α and SOD2 via SIRT1 and SIRT3, therefore increasing mitochondrial mass and function via PGC1α-activated mitochondrial biogenesis. This study also identified that increased levels of cyclic adenosine monophosphate (cAMP) can mediate the metabolic effects of PGC1α on mitochondrial function and biogenesis via the increased expression of PGC1α. These findings provide biochemical evidence that CoQ_10_ is capable of regulating mitochondrial activity by enhancing the activity of the cAMP-AMPK-SIRT1-PGC1α-pathway. Furthermore, at the inner mitochondrial membrane level, CoQ_10_ has been acknowledged as a modulator of mPTP, and has been demonstrated to prevent the opening of the mPTP in order to prevent ΔΨm collapse, therefore inhibiting mitochondrial-mediated apoptosis [147,148,149]. A summary of the neuroprotective effects of the bioactive quinones are outlined in Figure 10.

##### Pyrroloquinoline Quinone (PQQ)

PQQ, a natural redox cofactor, has been reported to stimulate mitochondrial biogenesis and has been associated with several health benefits due to its antioxidant properties, including improved mitochondrial function, energy utilization, and longevity, in addition to inhibiting apoptosis, providing protection from ROS, and improving neurologic functions in animal and cellular models [150,151,152]. A study carried out in mouse hepatocytes (Hepa1-6 cells) by Chowanadisai (2013) demonstrated that PQQ treatment was capable of increasing the mitochondrial content and stimulating mitochondrial biogenesis, indicated by an increased CS and cytochrome *c* activity, mtDNA content, and cellular oxygen respiration [151]. PQQ exposure was shown to promote phosphorylation of cAMP response element-binding protein (CREB), activating the PGC1α promoter, therefore increasing PGC1α mRNA transcription and protein expression, in comparison with the post-translation regulation of PGC1α by resveratrol. In addition, PQQ exposure to the Hepa1-6 cells was also found to increase the activation of NRF1 and 2. Moreover, the neuroprotective effects of PQQ were studied in a mouse rotenone-induced PD model, where PQQ was found to protect SH-SY5Y cells and dopaminergic neurons from neurotoxicity and to prevent mitochondrial dysfunction through the promotion of mitochondrial biogenesis, in addition to preventing motor deficits and reducing dopaminergic neuronal loss in the SNpc and dopamine depletion in the striatum [153]. PQQ has been shown to be a promising therapeutic candidate in animal models and cell cultures in order to ameliorate mitochondrial dysfunction through mitochondrial biogenesis induction, and this compound has therapeutic potential, as PQQ is water-soluble and low dietary concentrations are easily absorbed in the intestines, in comparison with natural polyphenols, which are water-insoluble and have poor absorption [151].

##### Idebenone

Idebenone (2,3-dimethoxy-5-methyl-6-(10-hydroxydecyl)-1,4-benzoquinone) is a synthetic analogue of CoQ_10_ that is active in the CNS and is capable of crossing the BBB [154,155,156,157]. It is an important antioxidant that has demonstrated a neuroprotective role in several neurological disorders, including PD. The pharmacological function of idebenone is centred on the activation of the electron transfer system, in addition to the reduction of non-respiratory oxygen consumption after conversion to its reduced, ubiquinol form by the MRC complex I [154]. Idebenone has also been found to be successful at inhibiting oxidative processes, such as lipid peroxidation. In a study carried out by Yan et al. (2019), idebenone treatment (100 mg/kg) in mice was shown to improve MPTP-induced dopaminergic neuronal death and the neurological deficits associated with PD, in addition to diminishing the inflammatory response by reducing the expression of several proinflammatory cytokines in the striatum and substantia nigra [156]. Moreover, in vitro studies have also demonstrated that treatment with idebenone can protect against MPTP-induced motor dysfunction and reduce neuronal death in the substantia nigra and striatum. 

#### 4.1.3. PPARγ Agonists

Numerous animal and cellular models and clinical studies have shown that PPARγ-agonists may represent a novel therapy for the treatment of several neurodegenerative conditions [158]. Drugs that activate PPARγ, for example thiazolidinediones (TZDs), commonly used for the treatment of diabetes mellitus, may be a potential treatment for neurodegenerative disorders due to their antioxidant, anti-inflammatory, and anti-apoptotic properties [46]. PPARγ activation was found to upregulate mitochondrial biogenesis; increase antioxidant defenses, ΔΨm, and transcription factors, including PGC1α and Nrf1/2; and regulate autophagy. In a human neuroblastoma SH-SY5Y cell model, rosiglitazone—a TZD and PPARγ-agonist [159]—was shown to induce antioxidant enzymes, such as SOD and CAT, and protect against neuronal damage induced by acetaldehyde in order to prevent the increased generation of intracellular ROS and apoptotic cell death [160]. Promoters of these antioxidant enzymes contain a PPAR response element, therefore rosiglitazone may potentially regulate the expression of SOD and CAT to protect the cells from ROS-induced damage. This study also showed that in acetylaldehyde-treated cells, rosiglitazone regulates B-cell lymphoma 2 (Bcl-2) expression, a cellular protein with anti-apoptotic properties associated with mitochondrial membrane permeability stabilisation, thereby maintaining mitochondrial integrity by suppressing cytochrome *c* release and inhibiting apoptosis. Pioglitazone treatment—an alternative TZD—was found to increase mtDNA content, the expression of nuclear-encoded MRC subunit proteins, and complex I (NADH: ubiquinone oxidoreductase) and complex IV (cytochrome c oxidase) activities in the human neuron-like NT2 cell line [46,161]. Moreover, in rat cortical slices and astrocytes, the PPAγ ligand 15D-PGJ2 and rosiglitazone demonstrated a neuroprotective effect by inhibiting cerebral oxidative damage following repeated stress, in addition to replenishing cerebral ATP levels and preventing impaired glutamate uptake [162]. Pre-clinical studies in animal models of PD have shown that rosiglitazone protects dopaminergic neurons and reduces neuroinflammation [119]. However, these drugs are often associated with adverse outcomes, including increased risk of heart failure and obesity [158]. 

It has been reported in recent studies that decanoic acid (C10) can interact with PPARγ in order to stimulate mitochondrial biogenesis and increase the antioxidant status in several neurological disorders [163]. The medium chain triglyceride (MCT)-based ketogenic diet (KD) has been found to be an effective treatment for neurodegenerative diseases and IMDs, as C10 has been shown to improve mitochondrial function and energy metabolism in patients with mitochondrial disorders and in neuronal cell models of these diseases [164]. In a study by Hughes et al. (2014), SH-SY5Y cells and cultured human skin fibroblasts were exposed to 250 μM C10, which stimulated significant increases in CS activity, as well as PPARγ-dependent increases in neuronal mitochondrial content, as indicated by the increased MRC complex I and CAT activities [165]. Moreover, the incubation of HT22 hippocampal murine neurons with C10 and β-hydroxybutyrate (βHB)—a ketone body—resulted in the upregulation of the MRC in comparison with the control cells, indicated by significant elevations of the SIRT1 and MRC complex I + III and complex IV enzyme activities. The activity of the CS enzyme was also significantly elevated in the treated HT22 cells, suggesting an induction of mitochondrial biogenesis and a probable increase in mitochondrial function and mass. As C10 is a medium chain fatty acid, it is able to efficiently cross the BBB where it can be oxidised via mitochondrial β-oxidation in the brain, therefore C10 may be a beneficial treatment for patients with neurodegeneration [166]. However, co-supplementation of C10 and octanoic acid (C8) in the KD should be considered, as the presence of C8 enhances the effect of C10 by decreasing its metabolism via β-oxidation and therefore improving the efficacy of this potential treatment [163]. 

### 4.2. Targeting Nrf2-ARE Pathway

One way in which the cell regulates its endogenous antioxidant capacity is via the activation of the transcription factor, Nrf2 [25]. The overexpression of Nrf2 or small molecule Nrf2 activators have been demonstrated to protect the CNS from oxidative damage by increasing the endogenous antioxidant capacity of the brain, and there are several Nrf2 activators undergoing clinical research for their effect on neurodegeneration outlined in the review by Robledinos-Antón et al. (2019) [167]. Therefore, the Nrf2-ARE pathway may be a potential therapeutic target in order to prevent the progression of neurodegeneration in various neurodegenerative and metabolic disorders [25,168]. For the purpose of this section, we will describe two examples of activators of Nrf2. 

#### 4.2.1. Chrysin

Chrysin (5,7-dihydroxyflavone) is a bioactive flavonoid that is naturally found in fruits, vegetables, and medicinal plants [169]. It has several biological properties, including antioxidant, anti-inflammatory, anti-apoptotic, and neuroprotective effects [169,170]. Although the mechanisms of Nrf2 activation by chrysin are not yet well-understood, evidence has revealed that chrysin can increase the expression and transcriptional activity of Nrf2, which in turn promotes the upregulation of the Nrf2-ARE pathway, resulting in an elevation in the expression of several antioxidant and phase II detoxifying enzymes, including heme oxygenase-1 (HO-1), SOD, and CAT. Studies with Nrf2 KO mice models have reported a greater loss of dopamine transporter levels within the striatum following 1-methyl-4-phenyl-1,2,3,6,-tetrahydropyridine (MPTP) treatment, a pharmacologically induced model of PD, inducing neurotoxicity [171]. Mice astrocytes overexpressing Nrf2 were found to completely reverse dopaminergic neuronal loss in the substantia nigra associated with MPTP treatment, suggesting that the activation of the Nrf2-ARE pathway in astrocytes may be sufficient to protect neurons from MPTP-induced neurotoxicity. This may alleviate the effects of OS in neurodegeneration, as neurons frequently depend on astrocytes for protection against OS due to increased activation of Nrf2-ARE-dependent gene expression in the latter cells [25,168]. A study undertaken by Thangarajan et al. (2016) discovered that the oral administration of chrysin (50 mg/kg) for 14 days in rats significantly improved behavioural deficits; regulated MRC complex I, II, IV, and V activities; diminished OS markers, including lipid peroxidation and protein carbonyls; inhibited apoptosis and significantly elevated the cellular antioxidant status (SOD, CAT, and GSH) in the striatal mitochondria [172]. This suggested that chrysin treatment may be beneficial for enhancing the survival of striatal neurons. However, similar to other natural flavonoids, chrysin has a low bioavailability, poor absorption, and is rapidly metabolised, therefore a combined pre-treatment of chrysin and protocatechuic acid (PCA)—another polyphenolic compound—has been studied in several in vivo and in vitro models. PCA has been shown to enhance the neuroprotective effects of chrysin in PC12 cells by reducing the MPTP-induced loss of SNpc dopaminergic neurons in mice, resulting in a greater cell viability, decreased lactate dehydrogenase (LDH) release, and inhibition of lipid peroxidation, in addition to inhibiting neuronal loss, increasing striatum dopamine levels, and restoring behavioural deficits in PD animal models [170,172]. Chrysin and PCA treatment was also found to significantly increase the transcriptional activity of Nrf2-responsive promoters and Nrf2 protein expression, triggering downstream regulation of the antioxidant enzymes, including increased intracellular SOD and CAT activity, in addition to a reduction in MDA levels, a marker of lipid peroxidation [173]. These findings indicate a potential neuroprotective role of chrysin combination therapy by reducing OS and providing enhanced antioxidant neuroprotection in neurodegenerative disorders. 

#### 4.2.2. Fumaric Acid Esters

Fumaric acid esters (FAEs) are a class of electrophiles that have been found to be neuroprotective in animal and cellular models of neurodegeneration via the activation of the Nrf2-ARE pathway [25]. In vivo evidence has demonstrated that dimethylfumarate (DMF), a pharmacological Nrf2 activator that has been shown to successfully reduce OS in multiple sclerosis (MS), may also have beneficial effects in PD [25,170,174]. In vitro studies have shown that DMF and its bioactive metabolite, monomethylfumarate (MMF), covalently modify Keap1 resulting in the accumulation of Nrf2, leading to the upregulation of Nrf2-dependent genes [175,176]. In a study undertaken by Lastres-Becker et al. (2016), a mice model of α-synucleinopathy indicated that DMF can target Nrf2 at the basal ganglia and can protect nigral dopaminergic neurons from α-synuclein toxicity, thus preventing neuronal cell death [176]. Daily oral DMF administration in this mouse model was also demonstrated to reduce astrocytosis and microgliosis, as well as prevent behavioral deficits. However, this protection was found to be absent in Nrf2 KO mice, indicating that DMF targets Nrf2 in order for DMF to exert its neuroprotective properties. Moreover, DMF and MMF treatment in animal and primary cultures of CNS cells have been reported to increase the nuclear levels of Nrf2, resulting in increases in the cellular redox potential, GSH and ATP levels, and ΔΨm [175]. In neurons and astrocytes, DMF and MMF were found to be cytoprotective against OS-induced cellular injury and death, in addition to significantly improving cell viability via upregulation of the Nrf2-dependent antioxidant response, indicated by the intracellular regulation of GSH [176,177,178]. Other studies have demonstrated improvements in mitochondrial function following DMF and MMF treatment by upregulating mitochondrial biogenesis in an Nrf2-dependent manner [175].

### 4.3. Targeting Impaired Mitophagy and/or Autophagy

Evolving studies have provided new insights into how dysfunctional autophagy may result in mitochondrial dysfunction and cellular stress, as well as the association between mitophagy and cellular OS in the development and progression of neurodegeneration [179]. Therefore, impaired autophagic function may be a potential mitochondria-targeted therapeutic in order to prevent mitochondrial dysfunction and improve neurodegenerative aetiology in neurodegenerative diseases. Several therapeutic targets and compounds have been identified that are known to stimulate autophagy/mitophagy. 

#### 4.3.1. Deubiquitinases

The ubiquitin-proteasome system (UPS) is often disturbed in many neurodegenerative diseases, demonstrating a potential therapeutic target [26]. A potential key target within this system are the deubiquitinating enzymes (DUBs), which function to catalyse the cleavage of ubiquitin from target proteins in addition to regulating proteasome activity [26,180,181]. Several DUBs have been implicated in the progression of neurodegeneration in a number of animal and cellular models, and are interesting novel therapeutic targets against neurodegeneration as they have been found to induce mitophagy in the absence of parkin/PINK1, in order to prompt mitochondrial clearance. Firstly, modulation of the DUB ubiquitin-specific peptidase 14 (USP14) has been demonstrated to affect mitophagy via induction of autophagy and by enhancing proteasome-mediated mitochondrial membrane rupture [26]. USP14 is an attractive target as it can be specifically inhibited by IU1, a non-toxic small molecule that binds to the USP14 catalytic domain to prevent the binding of the substrate to the enzyme, resulting in an increased proteasome activity and enhanced mitophagy [26,182]. In an in vivo Drosophila PD model with a PINK1/parkin mutant phenotype, USP14 knockdown or pharmacological inhibition restored mitochondrial function and increased mitochondrial clearance in neuronal cells [26]. USP14 inhibition has also been shown to increase autophagosome/autolysosome formation and enhances mitophagy in a human dopaminergic cell line [183,184]. USP30 inhibition is another DUB that may be an attractive target, as USP30 KO has been shown to increase mitophagy in human cell lines overexpressing parkin [185]. USP30 is localised to the OMM and USP30 overexpression was found to prevent parkin-driven mitophagy. However, reducing the activity of USP30 enhanced the mitochondrial degradation in neurons, in addition to rescuing defective mitophagy and improving mitochondrial integrity in PINK1 and parkin-deficient *Drosophila* models. USP30 KO in *Drosophila* was also found to protect dopaminergic neurons, therefore improving defective dopamine levels, motor function, and organism survival.

#### 4.3.2. Trehalose

Trehalose, a natural, non-toxic disaccharide, has been reported to function as a mammalian target of a rapamycin (mTOR)-independent inducer of autophagy and can protect the cell from several environmental stressors [186,187]. It has been demonstrated to relieve mitochondrial and neuronal cell damage through its antioxidative and mitophagy-inducing effects, as well as displaying anti-apoptotic effects through ameliorating mitochondrial dysfunction and restoring the autophagic flux. The effects of trehalose were analysed in a mouse model of manganese-induced mitochondrial dysfunction and neuronal cell damage by Liu et al. (2019), where trehalose was shown to inhibit OS-induced mitochondrial dysfunction, such as ATP depletion and reduced ΔΨm, via induction of mitophagy, demonstrating a protective role in damaged neurons [188]. Moreover, trehalose is able to activate transcription factor EB (TFEB), the master regulator of the autosomal-lysosomal degradation pathway, via Akt inhibition—a negative regulator of TFEB—which has been widely demonstrated to ameliorate the pathology in LSDs and neurodegenerative disorders [189]. The clearance of protein aggregates was augmented in trehalose-treated PC12 cells expressing mutant α-synuclein, and parkin deficient mice given trehalose-treated drinking water were shown to have increased autophagy and GSH levels together with a reduction in dopaminergic cell death [186,190]. Trehalose has been shown to target the brain when administrated orally in mice, slowing disease progression and extending survival in MPS IIIB mice models, in addition to improving behavioural disturbances and neuropathological features of this LSD [191]. This treatment was also shown to promote autophagic flux in the cortex and cerebellum of MPS IIIB mice, therefore increasing the clearance of autophagic vacuoles. 

A summary of the therapeutic strategies capable of alleviating mitochondrial dysfunction in neurodegeneration are outlined in Supplementary Table S1.

## 5. Conclusions

Mitochondrial dysfunction has been implicated in the pathophysiology of several inherited and non-communicable disorders that typically present neurologically, and has been suggested as a key event leading to the development of neurodegeneration. Thus, the development of mitochondrial-targeted therapies represents important novel therapeutic targets that have the potential to restore mitochondrial function, while simultaneously maintaining neuronal cell integrity and function, promoting neuronal cell survival and preventing neurodegeneration. The therapies discussed in this review are associated with targeting mitochondrial molecular pathways, in order to alleviate mitochondrial dysfunction and CNS disturbances, in addition to augmenting the antioxidant capacity of the cell. In general, these therapies demonstrated significant improvements in mitochondrial function and neuronal cell viability in several human, animal, and cellular models, indicating that therapeutic approaches that target mitochondrial-associated molecular pathways may be beneficial for preventing mitochondrial dysfunction in neurodegeneration. Although numerous studies have identified the favourable effects of these candidate therapies, a number of issues associated with BBB transport still remain, such as the low bioavailability and solubility of some of these compounds, which may affect their therapeutic efficacy. Therefore, before these therapies can be offered as potential therapeutic agents, further research is required in order to determine the efficacy and safety of these candidate therapies, particularly for inherited neurometabolic disorders. 

## Data Availability

Not applicable.

## Supplementary Materials

The following are available online at https://www.mdpi.com/article/10.3390/ijms222111444/s1.

## Abbreviations

AdoCbl	5′-deoxyadenosylcobalamin
Akt	Protein kinase B
ARE	Antioxidant-responsive element
ADP	Adenosine diphosphate
AMPK	AMP-dependent kinase
ATP	Adenosine triphosphate
AZ	Alzheimer’s disease
BBB	Blood-brain-barrier
Bcl-2	B-cell lymphoma 2
C8	Octanoic acid
C10	Decanoic acid
cAMP	Cyclic adenosine monophosphate
CAT	Catalase
CCCP	Carbonyl cyanide-m-chlorophenyl-hydrazine
CNS	Central nervous system
CoQ_10_	Coenzyme Q_10_
Complex I	NADH: ubiquinone oxidoreductase
Complex II	Succinate dehydrogenase
Complex III	Ubiquinol-cytochrome c oxidoreductase
Complex IV	Cytochrome c oxidase
Complex V	ATP synthase
CREB	cAMP response element-binding protein
CS	Citrate synthase
CSF	Cerebrospinal fluid
CβE	Conduritol-β-epoxide
DMF	Dimethyl fumarate
DUB	Deubiquitinase
ETC	Electron transport chain
FAE	Fumaric acid esters
FCCP	4-(trifluoromethoxy)phenylhydrazone
GCase	Glucocerebrosidase
GD	Gaucher disease
GPx	Glutathione peroxidase
GSH	Glutathione
H_2_O_2_	Hydrogen peroxide
HNE	4-hydroxyl-2-nonenal
HO-1	Heme oxygenase-1
IMD	Inherited metabolic disorder
iPSC	Induced pluripotent stem cell
KD	Ketogenic diet
Keap1	Kelch-like ECH-associated protein 1
KO	Knockout
LDH	Lactate dehydrogenase
LSD	Lysosomal storage disorder
MA	Malonic acid
MCA	2-methylcitrate
MCT	Medium chain triglyceride
MEF2C	Myocyte enhancer factor 2
MMA	Methylmalonic acid
MMAemia	Methylmalonic acidaemia
MMF	Monomethylfumarate
MMUT	L-methylmalonyl-CoA mutase
MnSOD	Manganese superoxide dismutase
MPS	Mucopolysaccharidosis
MPTP	1-methyl-4-phenyl-1,2,3,6,-tetrahydropyridine
mPTP	Mitochondrial permeability transition pore
MRC	Mitochondrial respiratory chain
mRNA	Messenger RNA
MS	Multiple sclerosis
mtDNA	Mitochondrial DNA
NAD^+^	Nicotinamide adenine dinucleotide
NCD	Non-communicable disease
NPC	Niemann Pick Type C
Nrf1	Nuclear respiratory factor 1
Nrf2	Nuclear factor erythroid 2-related factor
NQO1	NADH quinone oxidoreductase
OMM	Outer mitochondrial membrane
OS	Oxidative stress
OXPHOS	Oxidative phosphorylation
PCA	Protocatechuic acid
PD	Parkinson’s disease
PGC1α	Peroxisome proliferator-activated receptor gamma coactivator 1-alpha
PINK1	PTEN-induced kinase 1
PPARγ	Peroxisome proliferator-activated receptor gamma
PPQ	Pyrroloquinoline quinone
PTEN	Phosphatase and tensin homolog
ROS	Reactive oxygen species
RNS	Reactive nitrogen species
SAM	Senescence-accelerated mouse
Ser/Thr	Serine/threonine
SIRT	Silent mating type information regulation 2 homolog
SNpc	Substantia nigra pars compacta
SOD	Superoxide dismutase
TCA cycle	Tricarboxylic acid cycle
TFEB	Transcription factor EB
TZD	Thiazolidinedione
UPS	Ubiquitin-proteasome system
USP	Ubiquitin-specific peptidase
VDAC1	Voltage-dependent anion-selective channel 1
ΔΨm	Mitochondrial membrane potential
βHB	β-hydroxybutyrate
