# Supplementary material for: Targetable Pathways for Alleviating Mitochondrial Dysfunction in Neurodegeneration of Metabolic and Non-Metabolic Diseases
Source: Int J Mol Sci. 2021 Oct 23;22(21):11444. doi: 10.3390/ijms222111444 (PMC8583882; doi:10.3390/ijms222111444)
Supplement: Supplementary file 1 [file ijms-22-11444-s001.zip › ijms-1354736-supplementary.pdf]

Treatment		Targetable pathway	Method of action	Effectiveness and Considerations	Reference(s)
Natural polyphenols	Resveratrol	Induce mitochondrial biogenesis	Interaction with SIRT1 and AMPK via PGC1α	• Stimulates SIRT1-dependent upregulation of antioxidants in brain (SOD1+2, MnSOD, GPx, CAT).<br>• Promotes neuronal survival by inhibiting oxidative stress and preventing neuroinflammation.<br>• Improves oxidative function, increases mitochondrial biogenesis, MRC complex I and CS activity, ATP production, and decreases [lactate].<br>• Rapidly metabolised, low solubility and bioavailability.	[16] [46] [54] [105] [133] [138] [141] [186]
	Curcumin		Interaction with multiple signalling pathways (AMPK, SIRT1, Nrf2)	• Favourable absorption, bioavailability and long half-life in the CNS.<br>• Amphiphilic nature makes it beneficial in neurologic disease patients due to high % of lipids in brain.<br>• Improves O_2 consumption rates, increases cell viability and ΔΨm in PINK1-deficient cells.<br>• Increases dopamine levels, reduces cytotoxicity and cell viability in SNpc neurons.	[35] [50] [106] [117] [187]
Bioactive quinones	CoQ_{10}		Regulates mitochondrial activity via cAMP-AMPK-SIRT1-PGC1α pathway	• Improves cognitive function, facilitates ATP synthesis and upregulates mitochondrial function.	[2] [3] [30] [78] [86] [121] [131] [142] [189]

				• Reduces ROS generation, preserves ΔΨm, increases [ATP], prevents mitochondrial membrane collapse.<br>• Increases PGC1α signalling.<br>• Modulates mPTP inhibiting apoptosis.<br>• Absorption and bioavailability are major determinants of the efficacy of CoQ_{10} dietary supplementation.<br>• Higher plasma concentrations are required in neurodegenerative diseases to produce a clinical response – large molecular weight and hydrophobicity.	
	PQQ		Promotes phosphorylation of CREB, activating the PGC1α promoter, increasing PGC1α mRNA transcription and protein expression	• Improves mitochondrial function, energy utilisation and longevity.<br>• Inhibits apoptosis.<br>• Provides protection from ROS.<br>• Improves neurologic function and protects neurons from neurotoxicity.<br>• Increases activation of NRF1+2.<br>• Water-soluble and easily absorbed at low dietary concentrations.	[74] [153]
	Idebenone		Directly transfers electrons to MRC complex III, bypassing MRC complex I and restoring cellular [ATP].	• Idebenone is rapidly absorbed following oral administration.<br>• Poor water solubility and high lipophilicity which may impair its oral bioavailability.<br>• Active in the CNS and capable of crossing the BBB.<br>• Capable of inhibiting lipid peroxidation.<br>• Improve and protect against MPTP-induced neuronal death.	[154] [155] [156] [157]

				• Reduce PD associated neurological deficits.	
PPARγ agonists	Rosiglitazone		Selective binding and activation of PPARγ	• Upregulate mitochondrial biogenesis, increase antioxidant defences (CAT, SOD) and transcription factors (PGC1α, NRF1+2) and regulates autophagy.<br>• Regulate Bcl-2 expression to maintain mitochondrial integrity by inhibiting apoptosis and cytochrome *c* release.<br>• Inhibit cerebral oxidative damage and replenish ATP levels.<br>• Associated with adverse outcomes – increased risk of heart failure and obesity.	[43] [53] [159] [160] [161] [162]
	Pioglitazone		Selective activation of PPARγ 1+2 and PPARα		
	Decanoic acid (C10)		Direct ligand of PPARγ. C10 binds and partially activates PPARγ.	• Improves mitochondrial function and energy metabolism.<br>• Increases neuronal mitochondrial content indicated by increasing MRC complex I and CAT activities.<br>• A medium chain fatty acid – can efficiently cross the BBB.<br>• Co-supplementation of C10 and C8 improves efficacy of C10 by reducing C10 metabolism via β-oxidation.	[46] [94] [103] [113]
Chrysin			Increases expression and transcriptional activity of Nrf2, promoting upregulation of Nrf2-ARE pathway	• Overexpression of Nrf2, reverses dopaminergic neuronal loss in substantia nigra.<br>• Oral supplementation of chrysin improves behavioural deficits, regulates MRC complex I, II, IV and V activities, diminishes oxidative stress markers, inhibits apoptosis	[54] [129] [170]

		Activate Nrf2-ARE pathway		and increases cellular antioxidant status (SOD, CAT, GSH).<br>• Low bioavailability, poor absorption and rapid metabolism.<br>• Combined treatment with PCA can improve neuroprotective effects of chrysin and reduce oxidative stress.	
Fumaric acid esters (FAEs)	DMF		Oxidises Keap-1 sulfhydryl (-SH) groups, resulting in dissociation of Keap-1 from Nrf2, leading to Nrf2 migration to the nucleus and inducing upregulation of Nrf2-dependent genes	• Target Nrf2 at basal ganglia and protect nigral dopaminergic neurons from α-synuclein toxicity.<br>• Increase nuclear levels of Nrf2 to increase cellular redox potential, GSH, ATP levels and $\Delta\Psi m$.<br>• Cytoprotective against oxidative stress-induced cellular injury and death.<br>• Improve cell viability via upregulation of Nrf2-dependent antioxidant response.<br>• Each FAE exhibits different biodistribution in neurodegenerative disease.	[34] [122] [137] [174] [175] [178]
Deubiquitinases (DUBs)	USP14		Acts as a proteasome-associated DUB, releasing ubiquitin from the proteasome and targeting ubiquitinated proteins.	• Induce mitophagy in absence of PINK1/parkin.<br>• Pharmacological USP14 inhibition and/or KO restores mitochondrial function and increases mitochondrial clearance in neuronal cells.<br>• USP30 KO rescues defective mitophagy and improves mitochondrial integrity in PINK1/parkin-deficient cells.	[26] [71] [181] [183] [184] [185]

	USP30	Induces mitophagy/ autophagy	Negatively regulates mitophagy by removing ubiquitin from substrates ubiquitinated by Parkin	• Difficult to specifically target one DUB without affecting other DUBs due to several DUBs sharing the same structure and properties.	
Trehalose			Activates TFEB via Akt inhibition	• Inhibits oxidative stress-induced mitochondrial dysfunction via induction of mitophagy.<br>• Induces clearance of protein aggregates.<br>• Increases autophagy and GSH levels.<br>• Reduces dopaminergic cell death.<br>• Oral administration can target the brain – slowing down disease progression and improving behavioural disturbances and neuropathological features in LSDs.	[74] [87] [107] [149] [162]

Table S1. Summary of therapeutic strategies capable of alleviating mitochondrial dysfunction in neurodegeneration. The pathway targeted, mechanism of action and effectiveness and considerations of each of the therapeutic candidates have been considered.

SIRT1 – sirtuin 1; AMPK – AMP-dependent kinase; PGC1α - Peroxisome proliferator-activated receptor gamma coactivator 1-alpha; SOD – superoxide dismutase; MnSOD – manganese superoxide dismutase; GPx – glutathione peroxidase; GSH – glutathione; CAT - catalase; OS – oxidative stress; MRC – mitochondrial respiratory chain; CS – citrate synthase; ATP – adenosine triphosphate; Nrf2 - Nuclear factor erythroid 2-related factor; CNS – central nervous system; ΔΨm – mitochondrial membrane potential; PINK1 – PTEN-induced kinase 1; SNpc – Substantia nigra pars compacta; cAMP – cyclic adenosine monophosphate; ROS – reactive oxygen species; CoQ_{10} - Coenzyme Q_{10}; PQQ - Pyrroloquinoline quinone; CREB – cAMP response element-binding protein; mRNA – messenger RNA; NRF1 - Nuclear respiratory factor 1; PPARγ - Peroxisome proliferator-activated receptor gamma; Bcl-2 - B-cell lymphoma 2; TZDs – Thiazolidinediones; BBB – blood brain barrier; C8 – octanoic acid; C10 – decanoic acid; ARE – antioxidant-responsive element; PCA - Protocatechuic acid; Keap1 - Kelch-like ECH-associated protein 1; FAEs – fumaric acid esters; DUBs – Deubiquitinases – TFEB - Transcription factor EB; Akt – Protein kinase B; USP14/30 - Ubiquitin-specific peptidase 14/30; KO – knockout; LSDs – lysosomal storage disorders.
